# The Effect of Dietary Interventions on Hypertriglyceridemia: From Public Health to Molecular Nutrition Evidence

**DOI:** 10.3390/nu14051104

**Published:** 2022-03-05

**Authors:** Karla Paulina Luna-Castillo, Xochitl Citlalli Olivares-Ochoa, Rocío Guadalupe Hernández-Ruiz, Iris Monserrat Llamas-Covarrubias, Saraí Citlalic Rodríguez-Reyes, Alejandra Betancourt-Núñez, Barbara Vizmanos, Erika Martínez-López, José Francisco Muñoz-Valle, Fabiola Márquez-Sandoval, Andres López-Quintero

**Affiliations:** 1 Doctorado en Ciencias de la Nutrición Traslacional, Centro Universitario de Ciencias de la Salud (CUCS), Universidad de Guadalajara (UdeG), Guadalajara 44340, Jalisco, Mexico; karla.luna3337@alumnos.udg.mx (K.P.L.-C.); xochitl.olivares2305@alumnos.udg.mx (X.C.O.-O.); rocio.hernandez9558@alumnos.udg.mx (R.G.H.-R.); iris.llamas@academicos.udg.mx (I.M.L.-C.); citlalic.rodriguez@academicos.udg.mx (S.C.R.-R.); alejandra.bnunez@academicos.udg.mx (A.B.-N.); bvizmanos@yahoo.com.mx (B.V.); erika.martinez@academicos.udg.mx (E.M.-L.); drjosefranciscomv@cucs.udg.mx (J.F.M.-V.); 2 Instituto de Nutrigenética y Nutrigenómica Traslacional, CUCS, UdeG, Guadalajara 44340, Jalisco, Mexico; 3 Instituto de Investigación en Ciencias Biomédicas, CUCS, UdeG, Guadalajara 44340, Jalisco, Mexico

**Keywords:** dietary interventions, triglycerides, hypertriglyceridemia, clinical trial, molecular nutrition

## Abstract

Approximately 25–50% of the population worldwide exhibits serum triglycerides (TG) (≥150 mg/dL) which are associated with an increased level of highly atherogenic remnant-like particles, non-alcoholic fatty liver disease, and pancreatitis risk. High serum TG levels could be related to cardiovascular disease, which is the most prevalent cause of mortality in Western countries. The etiology of hypertriglyceridemia (HTG) is multifactorial and can be classified as primary and secondary causes. Among the primary causes are genetic disorders. On the other hand, secondary causes of HTG comprise lifestyle factors, medical conditions, and drugs. Among lifestyle changes, adequate diets and nutrition are the initial steps to treat and prevent serum lipid alterations. Dietary intervention for HTG is recommended in order to modify the amount of macronutrients. Macronutrient distribution changes such as fat or protein, low-carbohydrate diets, and caloric restriction seem to be effective strategies in reducing TG levels. Particularly, the Mediterranean diet is the dietary pattern with the most consistent evidence for efficacy in HTG while the use of omega-3 supplements consumption is the dietary component with the highest number of randomized clinical trials (RCT) carried out with effective results on reducing TG. The aim of this review was to provide a better comprehension between human nutrition and lipid metabolism.

## 1. Introduction

Triglycerides (TG) are lipids conformed by three fatty acids attached to a glycerol molecule and their main function is to store energy as fat in the adipose tissue (AT). TG can be either ingested through diet or synthesized endogenously by the liver and AT [1]. When ingested, TG are transported within chylomicrons through the circulation in order to be delivered to extrahepatic tissues and finally the liver [2]. On the other hand, liver and AT are able to synthesize TG when there is enough energy and substrates in the organism, such as glucose. After lipid synthesis, the liver releases very low-density lipoproteins (VLDLs), and these lipoproteins perform a similar transport of TG as the chylomicron. Lipoproteins have different activities and functions depending on the combination of apolipoproteins (Apo) they contain; these Apos interact with receptors in the tissue to perform the lipid delivery [3]. Chylomicrons hold Apo-B48 and VLDL carries Apo-B100 in their structure when synthesized; both lipoproteins accept Apo-CII and Apo-E from HDL afterward when in circulation [4]. However, chylomicrons also contain Apo-A1, A2, A3, A5, and CIII, although their function is not well established [5].

TG from lipoproteins are stored and remain in lipid vesicles of the adipocytes with the participation of the lipoprotein lipase (LPL) until energy from lipids is required by the organism [6]. LPL is important to transform chylomicrons to remnants and VLDL to LDL. This enzyme is synthesized mainly in muscle (skeletal and cardiac) and in AT, promoting TG usage by muscle and lipid storage by AT. LPL actions can be reduced by physical inactivity and other factors producing hypertriglyceridemia (HTG) [2]. HTG is defined as fasting serum TG of 150 mg/dL (1.7 mmol/L) or superior and it is the most common form of dyslipidemia in the adult population [7]—between 15% to 20% of patients receiving medical practice are diagnosed with this condition [8]. Chronic elevated blood glucose promotes insulin resistance in AT and leads to the enhancement of intracellular hydrolysis of TG and releasing of free fatty acids into the circulation and the liver. Such a condition favors HTG [9,10]. HTG can be classified according to the serum level as mild, moderate and severe; the first two classifications are related to the accumulation of VLDL plus remnant particles, and severe HTG reflects the presence of fasting chylomicrons and excess of VLDL and remnants [11]. The etiology of HTG is multiple and can be classified as primary and secondary causes. Among the primary causes are genetic disorders such as familial chylomicronemia syndrome, familial HTG, familial combined hyperlipidemia, familial dysbetalipoproteinemia, and the inherited lipodystrophy syndromes that include congenital generalized lipodystrophy and familial partial lipodystrophy [7]. Most primary causes have a low prevalence except for the familial HTG and familial combined hyperlipidemia, which prevalence is between 1% to 10%. Secondary causes of HTG comprise lifestyle factors (alcohol, high saturated fat, high refined sugar and hypercaloric diets consumption and smoking), medical conditions (obesity, metabolic syndrome (MS), hypothyroidism, diabetes mellitus, renal disorders, Cushing’s syndrome, pregnancy, HIV-associated lipodystrophy), acromegaly, and drugs (thiazides, beta blockers, steroid hormones and others) [7,8,9,10,11,12,13]. Cardiovascular diseases (CVD) have been associated with HTG but in a controversial manner; nevertheless, TG-rich lipoproteins such as chylomicrons and VLDL are linked to CVD [14]. HTG is an important marker of increased levels of highly atherogenic remnant-like particles, non-alcoholic fatty liver, and pancreatitis risk. General treatment of HTG uses fibrates, statins, and omega-3 fatty acids combined with lifestyle changes [13,14]. Moreover, intravenous insulin infusion is used to reduce serum TG through the activation of LPL but mainly in patients with acute pancreatitis, which is an ailment often caused by HTG [7,13]. Among lifestyle changes, adequate diets and nutrition are the initial steps to treat and prevent serum lipid alterations. Dietary intervention for HTG is recommended in order to modify the amount of macronutrients, such as reducing dietary fat and simple carbohydrate intake [15]. This review explores the results of RCTs that have evaluated the effect of different diet interventions and nutrient compounds along with its impact on serum lipids; specifically, we focus on TG, aiming to provide a better comprehension between human nutrition and lipid metabolism. 

## 2. Materials and Methods

The literature search was performed using the PubMed database. The search strategy was carried out from January 2016 to December 2021. We used search terms with a combination of MESH terms and hand searching applicable in each search. The following search terms were divided into four sections: diet, food, nutrients and bioactive compounds, and molecular nutrition. The structured search strategies were in the following format: for diet “(((“Diet”[Mesh]) AND “Dyslipidemias”[Mesh]) OR “Hypertriglyceridemia”[Mesh]) AND “Adult”[Mesh]”, “(triglycerides lowering) AND (diet)”, for food (((((“Nuts”[Mesh]) AND “Dyslipidemias”[Mesh])) OR “Hypertriglyceridemia”[Mesh]) AND “Adult”[Mesh]) NOT “Pancreatitis”[Mesh], “Nuts AND triglycerides”, “flaxseed AND triglycerides”, “Soy AND triglycerides”, “(((((“Fruit”[Mesh] AND “Dyslipidemias”[Mesh])) OR “Hypertriglyceridemia”[Mesh]) AND “Adult”[Mesh]) NOT “Nuts”[Mesh] NOT Omega-3[Mesh])”, “((“Olive Oil”[Mesh]) AND “Triglycerides”[Mesh]) AND “Heart Disease Risk Factors”[Mesh]”, “olive oil” AND “triglycerides” AND “cardiovascular”, “((((“Spices”[Mesh]) AND “Dyslipidemias”[Mesh])) OR “Hypertriglyceridemia”[Mesh]) AND “Adult”[Mesh]”, “(Dyslipidemias) AND (Dairy Products)” “Spices AND triglycerides”, for nutrients and bioactive compounds “((((“Dietary Carbohydrates”[Mesh]) AND “Dyslipidemias”[Mesh])) OR “Hypertriglyceridemia”[Mesh]) AND “Adult”[Mesh]”, “Dietary Fiber” AND “triglycerides”, “Saturated Fatty Acids AND Dyslipidemias OR Hypertriglyceridemia AND Adult”, “(((“Fatty Acids, Omega-3”[Mesh]) AND “Dyslipidemias”[Mesh]) OR “Hypertriglyceridemia”[Mesh]) AND “Adult”[Mesh]”, “(Flavonoids intake) AND (triglycerides)”, “(flavonoids) AND (hypertriglyceridemia)” and for nutritional genomic “((cardiovascular risk) AND (genomic)) AND (diet)”, “((genetics polymorphisms) AND (cardiovascular risk)) AND (diet)”. All studies in English for the full manuscript were selected. The inclusion criteria for the study selection were: (1) RCT or pre-post intervention study; (2) Participants adults (>18 years aged) diagnosed with any of the following clinical conditions: overweight, obesity, T2DM, dyslipidemia, hypertension, or MS; (3) Dietary intervention with dietary patterns or diets, nuts, legumes, fruits, olive oil, dairy products spices, carbohydrates, dietary fiber, omega-3 fatty acid; (4) Primary or secondary outcomes included lipid profile, specifically fasting TG. Exclusion criteria involved a publication written in languages other than English, studies with participants having a diagnosis of chronic kidney disease, cancer, ovary polycystic syndrome, acute or chronic pancreatitis, HIV, postmenopausal women or menopausal women without some of the conditions mentioned previously, hemodialysis and sepsis, or involving children. Extracted data included bibliographic information, study design, participant characteristics, and main outcomes including relevant information about TG.

## 3. Dietary Interventions and Nutrient Compounds on the Improvements of Serum TG

### 3.1. Diet

Changes in lifestyle could be determinants for HTG management and treatment; one key factor is that dietary habits directed toward adherence to a healthy diet are beneficial in reducing cardiovascular risk [16]. In addition, the composition of the diet, total calories ingested, macronutrients, food quality, and adherence to dietary patterns may have an impact on lipid profile [16,17,18]. Several studies examined the relationship between diet and TG levels.

### 3.2. Caloric Restriction

Randomized clinical trials (RCT) have shown that adjustment of fat, carbohydrate and protein percentages, relative to total energy, as well as reduction of total energy intake, can reduce TG levels; however, the success of dietary intervention depends on factors such as the source of the fat or carbohydrate, as well as lifestyle modifications [19]. An RCT conducted by Johansen et al. in 2017 in subjects with obesity or overweight showed that following an isocaloric, but macronutrient-controlled diet, for only two days has positive effects on plasma TG levels (106.29 mg/dL (35.43–239.15) 1.2 mmol/L (0.4–2.7) vs. 97.43 mg/dL (35.43–230) 1.1 mmol/L (0.4–2.6) (*p* < 0.05)) compared to a regular diet without any nutritional counseling (70.86 mg/dL (35.43–115.15) 0.8 mmol/L (0.4–1.3) vs. 62 mg/dL (26.57–221.43) 0.7 mmol/L (0.3–2.5)). The authors highlight the benefit of macronutrient and energy control on TG levels [20].

In accordance with the foregoing caloric restriction and weight loss proposed for several decades as a strategy to reverse lifestyle-related HTG, it has been shown that a 5% weight loss improves TG levels and most cardiometabolic parameters [21]. Different studies have proposed calorie restriction as an approach to reduce TG levels, and some of them are described below. A randomized trial performed an intervention with two restrictive diets for 16 weeks. The participants, who were overweight or obese, were assigned to either a daily continuous energy restriction meal replacement program (DER) or a modified, alternate-day fasting meal replacement program (ADF + DER). There were significant reductions in TG at week 16 compared to baseline (*p* < 0.05), independent of treatment [17]. Positive effects on TG levels have also been reported in a crossover clinical trial. The participants were assigned to time-restricted feeding early (TRFe) (eating window between 8 a.m. and 5 p.m.) or time-restricted feeding delayed (TRFd) (eating window between 12 p.m. and 9 p.m.), separated by a 2-week washout phase. After 7 days, TRF reduced fasting TG (*p* = 0.003), but there was no effect of the feeding window on TG levels [22]. In this sense, a 3-week controlled body mass reduction program based on physical activity and a hypocaloric diet (25–30% less than total daily energy requirements) showed a decrease in TG (152.7 ± 87.3 mg/dL (1.72 ± 0.99 mmol/L) vs. 133.6 ± 58.1 mg/dL (1.51 ± 0.66 mmol/L)), potentially related to weight reduction, but without statistical significance (*p* = 0.1004) [23]. An unblinded clinical trial compared two types of calorie restriction patterns: continuous calorie restriction (CCR) vs. intermittent calorie restriction (ICR), and in 8 weeks both groups achieved a significant reduction in plasma TG (by 15.6 and 6.3% in the ICR and CCR groups, respectively) with no difference between treatment groups [18]. In addition, an RCT conducted by Tang et al. in 2020 evaluated the effectiveness of fasting-mimicking diets (FMD), which involve consuming 25% of energy needs on fast days and 125% of energy needs on alternating “feast days” [24]; this 4-month trial included meal replacement intervention for 3 months and normal diet for the last month, the intervention group consumed the meal replacement from Monday to Friday the second week of the month and had a normal food consumption the rest of the month, the control group consumed the meal replacement but fulfilled their total caloric requirements per day. The intervention showed a significant intergroup difference in TG levels 310.81 ± 41.63 mg/dL (3.51 ± 0.47 mmol/L) for the control group vs. 181.58 ± 47.83 mg/dL (2.05 ± 0.54 mmol/L) for the FMD group, in T2DM patients [25].

Finally, one RCT was designed to compare the effects of two dietary plans for weight loss with different nutritional characteristics; the participants were randomly assigned to the anti-inflammatory diet (AID) group or the control diet (CD) group. Foods such as olive oil, fruits, vegetables, nuts and seeds, garlic, onion, fermented dairy products, and fish have been shown to be potentially anti-inflammatory. After 6 weeks both groups showed a decrease in TG levels (−12.2% vs. 11.3%); in the group that followed the diet with anti-inflammatory foods the reduction was greater, 115.15 ± 79.7 mg/dL (1.3 ± 0.0.9 mmol/L) vs. 106.29 ± 49.6 mg/dL (1.2 ± 0.0.56 mmol/L) but not statistically significant. This highlights the importance of diet quality in addition to caloric restriction [26].

### 3.3. Fat

Some studies suggest that high-fat diets promote improvement in TG levels. In a crossover clinical trial conducted in 2020 by Petrisko et al. in adults with obesity, two diets low in carbohydrates and high in fat were compared, with one pattern including plant-origin foods and the other animal-origin foods. After four weeks of intervention, separated by four weeks of washout period between each regimen, the results showed that fasting serum TG concentrations were higher (*p* < 0.05) following 4 weeks of consuming the low-fat (LF) diet (118 ± 44.8 mg/dL (1.33 ± 0.51 mmol/L)) compared with the very low-carbohydrate diet including more animal products (VLCA) (93.1 ± 31.38 mg/dL (1.05 ± 0.35 mmol/L)) and very low-carbohydrate diet that is plant- and mushroom-based (VLCPM) (92.1 ± 37.0 mg/dL (1.04 ± 0.42 mmol/L)) [27]. Although in some studies high-fat diets have shown greater effectiveness in reducing TG, it is essential to consider the dietary context and other dietary habits that can be incorporated, for example avoiding the consumption of trans fats, sugary drinks, and highly processed foods. This was evidenced in a clinical trial conducted in Norway with obese male participants. An intervention was performed with a low-fat high-carbohydrate (LFHC) diet compared to a very high-fat low-carbohydrate (VHFLC) diet. After 12 weeks following the intervention, both groups showed a decrease in TG levels (128.43 ± 46.94 mg/dL (1.45 ± 0.53 mmol/L) vs. 92.12 ± 33.66 mg/dL (1.04 ± 0.38 mmol/L) in LFHC group and 134.63 ± 53.14 mg/dL (1.52 ± 0.60 mmol/L) vs. 87.69 ± 45.17 mg/dL (0.99 ± 0.51 mmol/L) in the VHFLC group), which could be related to habit change and the elimination of ultra-processed foods [28] (Table 1). An RCT sought to determine the appropriate distribution of macronutrients to improve TG response, and 3 diets were compared: LFHC; a low-carbohydrate and higher-fat diet; and a walnut-rich, high-fat, low-carbohydrate diet. All diets were accompanied with caloric restriction (500–1000 kcal). At 6 months, there were significant decreases in TG within all three diet arms in women who were insulin-resistant (*p* < 0.05). This suggests that weight loss related to lower caloric intake may positively influence TG levels for overweight or obese insulin-resistant women, presumably by reducing central fat deposition [29].

Walnuts are rich in polyunsaturated fatty acids (PUFA), including α-linolenic acid (ALA) and linoleic acid; they also contain phenolic acids, stilbenes, tocopherols, flavonoids, and melatonin. Due to their properties, walnuts could be a replacement strategy for saturated fatty acids (SFA) that are consistent with the recommendations to reduce cardiovascular risk. However, in a crossover study in subjects with overweight or obesity and/or elevated brachial blood pressure where SFA were replaced by PUFA from nuts or vegetable oils, no modification of serum TG was observed after 6 weeks [45]. In this regard, canola oil is a vegetable oil commonly used that is low in SFA, moderate in PUFA, and rich in monounsaturated fatty acids (MUFA) with cardioprotective qualities, and that is also available in a high-oleic acid variety (HOCO; 71% oleic acid). A randomized crossover clinical trial evaluated the difference between canola oil and variety HOCO intake on lipids compared to a control diet with a fatty acid composition characteristic of a Western diet. The three diets were identical in macronutrients but differed in fatty acid composition (Table 1). After intervention, all diets reduced TG levels (canola: 128.43 ± 3.54 mg/dL (1.45 ± 0.04 mmol/L) *p* = 0.0182; HOCO: 127.55 ± 3.54 mg/dL (1.44 ± 0.04 mmol/L) *p* = 0.0053; control: 124 ± 3.54 mg/dL (1.40 ± 0.04 mmol/L) *p* = 0.0002); however, there was a difference in Apos [38].

Within lipid metabolism, the magnitude of the postprandial response is determined by several factors. Among the dietary factors, the quality of fat can affect the response. In order to explore how the quality of fat influences the postprandial lipid response, in a crossover trial carried out in patients with familial hypercholesterolemia and normolipidemic controls, a meal with 40% PUFA or SFA was offered and venous blood samples were obtained at baseline (fasting) and at 2, 4, and 6 h after consumption of the meal. The TG levels reached their maximum point at 2 h after ingestion of a 40% PUFA meal and at 4 h after ingestion of a 40% SFA meal in both groups. No significant differences were found in TG levels between meals or groups [36]. The postprandial TG response has also been studied by Folwaczny et al., who in a crossover design administered a shake 80 g of fat with different types of fatty acids to hypertriglyceridemic patients. The postprandial TG elevation was lower in the medium chain fatty acids (MCFA) shake compared with the SFA shake (*p* = 0.03) and MUFA shake (*p* = 0.01) in hypertriglyceridemic patients, and these concentrations were significantly higher compared with the normolipidemic controls (SFA meal *p* = 0.019, MUFA *p* = 0.05) [49].

In addition to fatty acids, some oils such as rice bran oil, flaxseed oil, and sesame oil contain phytonutrients. These active compounds have the potential to lower blood TG concentrations. Haldar et al. mixed rice bran, flaxseed, and sesame oils with the purpose of improving blood lipid profile and cardiometabolic health. A mixture of these oils in borderline hypercholesterolemic was studied at a dose of 30 g per day for 8 weeks. Serum TG were lower (*p* < 0.0001) during the intervention period compared to baseline with all oils, but there were no statistical differences between the oil blending intervention and the refined olive oil intervention [46].

### 3.4. Carbohydrates

On the other hand, carbohydrate reduction has turned into an accepted strategy for HTG treatment. Some studies report low-carbohydrate diets to have better effectiveness in TG reduction than low-fat diets [34,44,48]. A clinical trial conducted in Japan in patients with T2DM showed that replacing normal bread with low-carbohydrate bread has positive effects on TG levels 2 h after food consumption, due to a decrease in the percentage of total carbohydrates per day (48.8% vs. 35.5%). At the same time, a low-carbohydrate diet decreases postprandial glucose and improves insulin resistance [41]. In an RCT, a low-carbohydrate diet was compared with a feeding plan established by the American Diabetes Association (ADA) in adults with obesity and type 2 diabetes mellitus (T2DM). After 32 weeks of intervention, it was shown that the low-carbohydrate diet diminished TG levels, estimated marginal mean ((EMM)–60.1 mg/dL (*p* = 0.01)) compared to ADA group (EMM –6.2 mg/dL) [34]. Similar data were mentioned in other RCT carried out on subjects with obesity, where healthy low-carbohydrate (HLC) and healthy low-fat (HLF) diets were analyzed. The authors also evaluated different consumption percentages of SFA. After 12 months, the HLC group presented lower triglycerides than the HLF group (100.6 ± 59.7 mg/dL (1.14 ± 0.67 mmol/L) vs. 119.2 ± 65.5 mg/dL (1.35 ± 0.74 mmol/L) (*p* = 0.003)). In the regression model it was shown that every 1% increase in SFA intake from baseline to 12 months in the HLC group resulted in a statistically significant decrease in TG (*p* = 0.04) without significant changes in LDL cholesterol or HDL cholesterol; however, results for TG were no longer statistically significant after further adjustment for 12 months change in carbohydrate intake (*p* = 0.09). This suggests that individuals who follow a low-carbohydrate diet and increase SFA consumption can decrease TG levels, provided that they focus on a high-quality diet and lower their intakes of refined carbohydrates [44].

Diet composition can have an impact on TG concentrations, and the evidence indicates that a diet high in refined starches and added sugars exacerbates the alteration in carbohydrate metabolism [32]. In a crossover clinical trial with overweight or obese subjects with HTG, the consumption of these nutrients was replaced by a mixture of protein and unsaturated fatty acids (UFA) during one of the intervention periods and a reduction in TG was observed at 3 weeks compared to the added sugar and refined starches period (*p* < 0.002) [32].

### 3.5. Protein

According to the percentage of protein intake, there is evidence that a high-protein diet is related to TG decrease. In a clinical trial conducted by Shah and colleagues in 2018, a comparison was made between a high-protein (HP) diet versus a high-monounsaturated-fat (HMF) diet in overweight and obese adults. The group on a high-protein diet showed a significant decrease in TG 3 h after food consumption compared to the high-fat diet. The responses were higher at 120 (*p* = 0.006) and 180 (*p* = 0.003) min during the HMF compared to the HP meal condition [16]. A high-protein meal may decrease TG levels through reduction in chylomicron formation due to decreased fat intake, faster chylomicron clearance by increasing LPL stimulation, effectiveness of lipid oxidation in the liver, and decreased lipid synthesis. The latter may be related to glucagon-like peptide-1, a hormone that is elevated after a high-protein meal compared to a high-fat meal [16,50,51].

Macronutrient partitioning is important but total energy is relevant as well. From this perspective, caloric restriction is proposed as a useful tool for decreasing TG levels, in synergy with an increase in protein intake. It has been shown that a protein intake of 30–35% is favorable coupled with a total calorie restriction [33,38]. According to the above, an RCT was conducted in adults with obesity, where two energy intake distributions were compared. The meal plan 1 was 15% protein, 30% fat, 55% carbohydrate, and 0.8 g/kg protein per day; meal plan 2 was 30% protein, 30% fat, 40% carbohydrate, and 1.2 g/kg protein per day. However, both groups had their calories restricted to 500 kcal and both groups decreased their total levels of TG, but the group with the hyperproteic diet showed a greater decrease (−17.3 ± 50.2 mg/dL (0.2 ± 0.57 mmol/L) vs. 11.5 ± 34.7 (0.13 ± 0.39 mmol/L)), with respect to meal plan 1 (0.8 g/kg protein) [43]. In addition, Mateo-Gallego and collaborators in 2017 conducted an RCT in obese women to compare three calorie-restricted diets with different protein percentages (Table 1). After 3 months TG levels were significantly lower in the 35%-protein group (high in animal protein) than in the 20%-protein group (94.1 ± 26.2 mg/dL (1.06 ± 0.3 mmol/L) (*p* = 0.0001) vs. 117 ± 74.1 mg/dL (1.32 ± 0.84 mmol/L)). Moreover, change in TG levels was negatively correlated with consumption of animal protein. The authors propose that an energy-restricted diet with 35% protein, mostly of animal origin, promotes a decrease in TG levels [33].

### 3.6. Food Combining and Nutritional Quality

The variety of foods with a positive effect on health is vast. It is widely recognized that fruits and vegetables are food groups that when included in the regular diet provide multiple bioactive compounds. Among those compounds are polyphenols, which have attracted increased interest in recent years due to their antioxidant and immunoregulatory capacity [52]. In this sense, it has been proposed that a diet high in polyphenols (fruit, vegetables, and omega-3), could have beneficial effects on the lipid profile. According to a study conducted by Della Pepa and collaborators it was determined that the consumption of polyphenols for 8 weeks decreases TG compared to a diet low in polyphenols (289.64 ± 194.86 mg/dL (3.27 ± 2.20 mmol/L) vs. 225.86 ± 189.55 mg/dL (2.55 ± 2.14 mmol/L)) in overweight or obese adults. There is also another dietary approach to TG lowering. It has been proposed to reduce the consumption of some foods such as red meat, sausages, and refined cereals that are associated with an increased risk of developing chronic degenerative diseases; however, interventions in this regard are limited [39]. Therefore, Kim and collaborators in 2017 developed an RCT where two diets were compared: one included some foods such as red meat and refined cereals (HMD) and the second was high in whole grains, nuts, legumes, and dairy, eliminating red meat and sausages (HWD). After 4 weeks of intervention, it was determined that TG concentrations were significantly higher after consumption of the HMD diet (84.15 ± 41.63 mg/dL (0.95 ± 0.47 mmol/L)) compared to the HWD diet (77.06.29 ± 38.09 mg/dL (0.87 ± 0.0.43 mmol/L)) [31]. As part of the same clinical trial, a second trial was conducted to measure postprandial TG levels after consuming two different meals, first red meat/refined grain meal (39.2 g carbohydrate, 6.4 g protein, 1.52 g fat), secondly dairy/chicken/nuts/wholegrain meal (34.6 g carbohydrate, 7.6 g protein, 2.56 g fat). Area under curve TG (AUC) did not differ between two meals (2.3, 2.4 mU/L/3 h for red meat/refined grain diet vs. 2.6, 1.8 mU/L/3 h for dairy/chicken/nuts/wholegrain diet, *p* = 0.172), but there was a small significant difference in incremental area under curve TG (iAUC) (−0.2, 0.5 mU/L/3 h for red meat/refined grain diet vs. 0.0, 0.4 mU/L/3 h for 12 of 17 dairy/chicken/nuts/wholegrain diet, *p* < 0.001). Consistent with the previous clinical trial, the HWD diet has a positive effect on both postprandial and serum TG levels after 4 weeks [30]. 

### 3.7. Dietary Patterns

Dietary patterns (DPs) tend to be heterogeneous between different countries, and many of them have been associated with a decrease in the prevalence of cardiovascular disease and a decrease in cardiometabolic risk. The effect of dietary patterns is determined by the quality of the component of foods and the effect they have on metabolism [37,53]. In this section we discuss different DPs included in these recommendations, such as the Korean diet, the Mediterranean diet, the Paleolithic diet, the Vegetarian diet, and the Portfolio diet.

A crossover clinical trial conducted in Korea compared three different diets—a typical Korean diet, a diet based on 2010 Dietary Guidelines for Americans (2010DGA), and a typical American diet—in which the percentage of macronutrient distribution was calculated and monitored (Table 1) and the total calories per day was adjusted for each individual. At the end of the study, the three diets showed a significant decrease in TG levels with respect to baseline, which demonstrates the importance of calorie and macronutrient percentage control, as previously reported. The change was greater for the Korean diet, and this could be related to the type of foods that were included, such as green leafy vegetables, fruits, whole grains, legumes, and fermented foods, as well as the low consumption of animal foods; however, it decreased HDL cholesterol levels (−3.32 ± 0.97 mg/dL) (−0.085 ± 0.025 mmol/L) (*p* = 0.001). In contrast to the 2010DGA diet, which not only decreased TG levels but also increased HDL levels (2.0 ± 0.98 mg/dL) (0.051 ± 0.025 mmol/L) (*p* = 0.044), this diet is based on American guidelines and is moderately low in carbohydrate and moderately high in fat composition compared to the Korean and typical American diets. In this sense, the authors conclude that by discretely decreasing the percentage of carbohydrates, eliminating refined sugars and foods high in saturated and trans fat, and increasing the consumption of fruit and vegetables, whole grains, low-fat dairy products, seafood, and vegetable oils, for 4 weeks, TG levels were decreased and HDL cholesterol levels were increased; it is therefore presented as an acceptable dietary strategy [47].

The Mediterranean dietary pattern incorporates foods such as Greek yogurt, low-fat cheese (ricotta, cottage cheese), extra virgin olive oil, fruits, leguminous vegetables, nuts and seeds, fish, vegetables, and whole grains, and decreases the consumption of sugar, processed foods, red meat, and sugary drinks. Because of this, the Mediterranean diet is high in antioxidants, fiber, vitamins and minerals, polyphenols, MUFA, and omega-3 polyunsaturated fats; it has therefore been proposed as a dietary model with great benefits for the prevention and treatment of diseases. The reduction in TG levels related to the Mediterranean diet has been evidenced in several studies [37,40,48]. In a crossover RCT conducted in Australia by Wade et al. in 2018, the effect of the Mediterranean diet on cardiovascular risk parameters was analyzed after 8 weeks of intervention and separated by 8 weeks of washout; the study showed a decrease in TG related to the Mediterranean diet compared to a low-fat diet, in adults with obesity [37]. However, evidence shows that the Mediterranean eating pattern has similar effects as a low-carbohydrate diet. In an RCT where the effect of the Mediterranean diet, in conjunction with a caloric restriction, on cardiometabolic risk factors was evaluated, after 24 months of intervention a significant decrease in TG was evidenced both in the group that followed the Mediterranean diet and in the group that followed a low-carbohydrate diet, from the first 6 months of intervention with both diets (*p* = 0.05) [48].

On the other hand, studies where the Mediterranean eating pattern and a low-carbohydrate diet have been evaluated in combination have not obtained significant results in the decrease of TG [40], which raises the possibility that both eating patterns could be effective separately for the treatment of cardiometabolic diseases.

The Paleolithic diet has its basis in anthropological research, where the type of diet in the Paleolithic period was proposed, according to the historical context and geographical location. The included foods are lean meat, poultry, fish, seafood, fruits, nuts, berries, seeds, vegetables, and drinking water; the “non-Paleolithic” foods were legumes, cereals, sugar, salt, processed foods, and dairy products. It has been proposed that this eating pattern holds health benefits, mainly because of the type of foods that are eliminated. In an RCT conducted by Mårtensson et al. in 2021, the effect of adherence to a Paleolithic diet on cardiometabolic risk factors was analyzed in overweight and obese subjects. A higher Paleolithic ratio at 12 weeks was associated with lower TG levels (*p* < 0.05), independently of weight loss [53].

Plant-based diets have gained popularity in the last decade, and they have been proposed as a dietary pattern with potential health benefits. However, a low-fat plant-based diet (i.e., a diet that includes whole grains, legumes, vegetables, and fruits, and approximately 7–15% total energy from fat) did not show any significant decrease in TG (*p* = 0.32) after 6 months of intervention compared to the control diet that had no restriction on total energy intake (Table 1). Although the program led to significant improvements in BMI, cholesterol, and other cardiovascular risk factors, there was no difference in TG levels. This may be due to the low fat intake established by the type of diet [35]. 

Finally, the Portfolio diet has been established as an alternative for the treatment of dyslipidemias. It is a plant-based dietary pattern that utilizes a “Portfolio” of four foods which can lower cholesterol. The four core food components of the Portfolio dietary pattern include (based on a 2000 kcal diet): 42 g nuts, 50 g plant protein, 20 g soluble fiber, and 2 g plant sterol [54]. Following this pattern, but modifying the foods included, an RCT was conducted to evaluate the effect of functional foods included in a portfolio dietary pattern through packets in dehydrated form ready to be dissolved in water. In the group following the Portfolio diet, it was found that they adhered to a combination of functional foods, including nopal, chia seeds, soy protein, and dehydrated inulin, and that the participants followed a reduced energy diet (−500 kcal). The placebo group received a dehydrated product with the same organoleptic characteristics as the intervention group (Table 1). A significant decrease in TG was found in the group that consumed the Portfolio diet (*p* < 0.01) in comparison with food used as placebo, after 3 months. This suggests that including functional foods in the regular diet has benefits on TG levels and other cardiometabolic markers in people with T2DM [42]. 

The evidence suggests that diet is a fundamental part of the treatment of HTG, and therefore that it decreases cardiovascular risk. On the one hand, macronutrient distribution and diets low in carbohydrates seem to be more effective in reducing TG levels, as long as the quality of the food is prioritized. It is important to decrease the dietary intake of simple carbohydrates and increase the consumption of legumes and vegetables significantly, which means increasing dietary fiber intake. Decreasing carbohydrate consumption from 20–50 g per day initially and then gradually increasing it (120–150 g/per day) is an intervention that has proven beneficial for TG levels. In this sense, avoiding the consumption of industrialized foods high in refined sugar and saturated and trans fats and including functional foods such as fruits, vegetables, olive oil, legumes, and oilseeds evidenced a significant decrease in TG as well. In addition, caloric restriction represents a common factor in many of the interventions reviewed. Reducing 500–1000 kcal is beneficial for lowering TG, as are fasting patterns to promote the restriction of total calories per day. On the other hand, adherence to dietary patterns such as the Mediterranean diet, the Portfolio diet, or the Paleolithic diet can significantly improve TG levels, related to the antioxidative and anti-inflammatory capacity of most of the foods that compose the aforementioned dietary patterns (Table 1).

## 4. Foods

### 4.1. Nuts and Flaxseed

The consumption of walnuts has been widely recommended in the population [55] due to several cardioprotective mechanisms. Walnuts are characterized by a high content of MUFA, PUFA, vegetable protein, dietary fiber, vitamins (vitamins E, K, folate, B1), minerals (magnesium, cooper, potassium, and selenium), and phenolic compounds such as carotenoids and phytosterols. These components have been predominantly related to health outcomes [56,57]. In this regard, the following paragraphs will present the scientific evidence on the effect of nut consumption on serum TG levels.

Many clinical trials have been performed to investigate the potential cardiovascular benefits of nuts [58,59,60,61,62,63]. A randomized, parallel-controlled trial involving 54 overweight and obese adults assigned to receive 42.5 g/day of mixed nuts (almonds, cashews, hazelnuts, pecans, brazil nuts, macadamia nuts, pistachios, walnuts, and peanuts) reported a significantly discreet reduction on TG after 8 weeks compared with baseline (110.25 ± 96.25 mg/dL (1.26 ± 1.10 mmol/L) vs. 101.5 ± 58.63 mg/dL (1.16 ± 0.67 mmol/L); *p* < 0.005). No significant differences were observed between the nut and pretzel groups (*p* = 0.655). This could be explained by the low baseline concentrations of the lipids [58]. Consistent with this finding, two additional interventions with nut-enriched diets showed significant decreases in TG after 1 year (−7.8 mg/dL ± 68.9 (−0.09 ± 0.79 mmol/L); *p* = 0.020) [60] and 8 weeks post intervention (−39.7 (−60.8, −18.3 mg/dL) (−0.45 (−0.69, −0.21 mmol/L); *p* < 0.05) [59], respectively. Furthermore, two-arm, parallel group randomized dietary intervention in 128 overweight and obese adults with a diet enriched with almonds for 12 weeks reported a significant reduction in serum levels of TG compared with the control group (intervention group (IG): 112.88 ± 6.13 mg/dL (1.29 ± 0.07 mmol/L) vs. 100.62 ± 5.25 mg/dL (1.15 ± 0.06 mmol/L); control group (CG): 101.5 ± 0.61 mg/dL (1.16 ± 0.007 mmol/L) vs. 103.25 ± 0.53 mg/dL (1.18 ± 0.006 mmol/L); *p* = 0.008) [61]. This observation was confirmed in two studies with single-type nut interventions, specifically with peanut and almond. The first one was an RCT in 15 overweight and obese men which evaluated an acute intervention with 85 g of peanut shake per visit for 10 days. It demonstrates a clear reduction in TG after 120 min (16,528.75 ± 1697.5 mg/dL (188.9 ± 19.4 mmol/L) vs. 17,281.25 ± 1811.25 mg/dL (197.5 ± 20.7mmol/L)) and 240 min (16,616.25 ± 2126.25 mg/dL (189.9 ± 24.3mmol/L) vs. 17,263.75 ± 1610 mg/dL (197.3 ± 18.4 mmol/L)) with peanut shake compared with control (*p* < 0.005). These results suggest that peanut consumption could protect against endothelial and oxidative damage. This cardioprotective effect could be related to the high content of L-arginine, which is an important precursor of nitric oxide synthesis [62]. The second one was a study in 50 T2DM subjects in which 20% of total energy of fat was replaced with raw almonds during 24 weeks, which showed a decrease in TG after intervention compared with baseline (170.5 (59.49 mg/dL) 1.94 (0.68 mmol/L) vs. 149.5 (72.49 mg/dL) 1.7 (0.83 mmol/L); *p* < 0.004) [63].

Other authors reported that whole flaxseed or products with flaxseed or flaxseed oil could improve TG concentration [46,64,65,66]. In 2020, a double-blind randomized trial in 53 overweight and obese patients found an improvement in TG after 60 days of intervention with 100 g/day of biscuits with flaxseed meal (105 ± 51.63 mg/dL (20 ± 0.59 mmol/L) vs. 101.5 ± 55.13 mg/dL (1.16 ± 0.63 mmol/L); *p* < 0.005). This reduction was significantly different compared to the control group (*p* < 0.05). The authors reported that the possible mechanism by which flaxseed meal reduces TG is that flaxseed fiber reduces fat absorption by binding to bile salts (cholic acid) and increasing fecal lipids in the intestine. Biscuit with flaxseed meal also increased LPL concentration, which explains the degradation of TG and consequently the improvement in serum levels [64]. In addition, Haldar et al. evaluated the effect of 30 g/day of refined rice bran, flaxseed, and sesame seed oil in a parallel randomized controlled trial involving 143 hypercholesterolemic subjects and showed a significant attenuation in TG (−10.3%; *p* < 0.001) after 8 weeks [46].

Furthermore, a consumption of 30 g/day of whole flaxseed powder appears to have a greater effect on TG reduction after 12 weeks (−66.0 ± 47.8 mg/dL (0.75 ± 0.54 mmol/L); *p* < 0.05) among different nut and flaxseed interventions, which also showed improvements in TG levels [46,59,60,61,63,65,67,68]. This study showed a significant reduction in TG after flaxseed intervention compared to baseline data (*p* < 0.001) and control group (*p* < 0.005). The possible mechanisms associated with the consumption of ALA-rich flaxseed and the regulation of lipid metabolism and improvements in TG are through the stimulation of ß-oxidation, the inhibition of fat acid synthesis and the activation of the peroxisome proliferator-activated receptor alpha (PPAR-α). Activation of PPAR-α has also been reported with the consumption of flavonoids such as hesperidin and the regulation of Apo-B100 synthesis [66].

Another randomized controlled trial involving 60 patients with MS showed a reduction in TG levels after consumption of 25 mL daily of flaxseed oil for 7 weeks compared with the control group (sunflower seed oil) (IG: −52.46 ± 74.32 mg/dL (−0.6 ± 0.85 mmol/L); *p* < 0.001 and CG: −53.46 ± 58.21 mg/dL (0.61 ± 0.66 mmol/L; *p* < 0.001)) [65]. Similar data were reported by Zibaeenezhad et al. in a randomized, double-blind, placebo-controlled clinical trial in 100 hyperlipidemic and T2DM patients with a supplementation of 15 mL of Persian walnut oil for 10 days. It indicated a significant decrease in TG by 15.04 % (*p* = 0.021) [67]. However, no significant differences were reported with a supplementation with 10 mL of almond oil two times daily in 99 hyperlipidemic adults (−4.3%, *p* = 0.441) [69]. By contrast, a randomized, double-blind, placebo-controlled clinical trial in 31 subjects with MS evaluated the effect of 10 mL of extra virgin brazil nut oil compared to 10 mL of soybean oil, showing negative effects on TG after brazil nut oil consumption for 30 days (68.6 ± 83.6 mg/dL (0.78 ± 0.95 mmol/L); *p* < 0.005), as opposed to the control group (soybean oil), which presented a reduction in TG by 4.5% (no significant difference). The results in the IG could be explained by the characteristics of the population composed of women of menopausal age, which has been related to alterations in the lipid profile. In addition, a reduction in the malondialdehyde/TG ratio suggests that Brazil nut oil could reduce lipid peroxidation. This is consistent with previous reports that PUFA was related to reductions in VLDL synthesis by enhancing lipid catabolism in peroxisomes and the decrease in TG due to the hydrolysis of Apo-B100 [68].

On the other hand, two clinical trials with an intervention of 28–64 g/day and 30 g/day with cashews found no significant difference in TG levels post intervention compared with the control group [70,71].

The evidence suggests that a diet enriched with nuts, mixed nuts, peanuts, and almonds could be useful for the reduction of TG, whereas cashews appear to have no effect on this biomarker. On the other hand, flaxseed products reduce TG in the range of −5 to −66 points. The incorporation of at least 30 g of mixed nuts, 30 g of whole flaxseed, and 25 mL of flaxseed oil into the diet could improve TG levels in subjects with cardiovascular risk (obesity, hypercholesterolemia, MS, and diabetes). It could also be a preventive strategy for cardiovascular disease risk reduction. By contrast, the findings reported for the intake of almond and pecan nut oil do not seem to be convincing for their recommendation (Table 2).

### 4.2. Legumes

Legumes have been promoted as an important part of sustainable plant-based diets due to their high nutritional value and low cost [72]. Soybeans are a good source of quality vegetable protein, soluble fiber, linoleic acid as the predominant fatty acid, vitamins (B1, B2, B3, B6), minerals (magnesium, potassium, phosphorus, iron, calcium), phytosterols, polyphenols, and isoflavones [72,73]. The aforementioned evidence would support the beneficial health effects of legumes in reducing CVD and promoting cardiometabolic health [74]. We focused on soybean, because as a result of the searches it was the only legume evaluated in RCT with primary or secondary TG outcomes.

The results of the lipid-lowering effects of soy products are still controversial and inconsistent among clinical trials. Changes on TG levels have differed among studies. A double-blind RCT assessed the effect of long consumption of 30 g/day soy milk fortified with phytosterols on serum lipids in hypercholesterolemic adults. The results showed a small reduction in serum TG concentrations after 6 months with fortified soy milk consumption (147 ± 89.25 mg/dL (1.68 ± 1.02 mmol/L) vs. 166.25 ± 85.75 (1.90 ± 0.98)) compared with baseline (without statistically significant) [75]. Years later, Oliveira et al. performed a double-blind, placebo-controlled crossover study with a supplementation of fortified soy milk with phytosterols, and an 8.3% reduction in TG levels was found, which was significantly different compared with the control group (*p* < 0.05) [76]. Greater effects have been observed with soy and pea shake interventions. A clinical trial study in obese people where two meals were replaced with a shake rich in soy protein and pea and soluble fiber showed a reduction of 11.5% in TG levels 8 weeks post intervention compared with baseline (98.7 ± 45.3 mg/dL (1.13 ± 0.52 mmol/L) vs. 111.5 ± 57.9 (1.27 0.66); *p* < 0.05) [77].

On the other hand, the intervention with soy extracts in capsules does not seem to improve serum TG concentrations. After 8 weeks with a lunasin-enriched soy extract capsule, in participants with cardiovascular risk factors showed no difference in TG. The TG were maintained at the same concentration (131.25 mg/dL (1.5 mmol/L) vs. 131.25 (1.5); *p* = 0.963) [78]. The differences between the studies could be explained by the variety of products used in the interventions.

The greatest effect of soy products for improving serum lipids has been observed in serum LDL cholesterol and total cholesterol concentrations [75,78]. However, their effects on serum TG are inconsistent due to differences in methodologies; further studies are therefore needed to define recommendations for the population (Table 3). 

### 4.3. Other Foods (Olive Oil, Low-Fat Milk, Spices, Fruits, and Green/Roasted Coffee Blend)

This section responds to the RCT resulting from the search that focuses on evaluating the consumption of food on TG, but there are not enough studies to generate a section referring to these.

According to some studies, olive oil is effective for the treatment and prevention of cardio metabolic diseases, but its effect must be accompanied by an adequate diet in macronutrients together with healthy habits. Olive oil alone did not produce significant changes in TG levels after 12 weeks of intervention [79]. Similar results were observed after 3 weeks of intervention with polyphenol-rich olive oil in a controlled crossover trial in hypercholesterolemic patients. There was no significant difference in change between baseline and post-intervention in all groups in TG [80].

Similar findings were obtained investigating the effects of low-fat milk consumption on cardiometabolic biomarkers, and no significant differences in changes in TG were found between the low-fat milk and control group (maintaining habitual diet) [81].

On the other hand, spices, or a combination of them, have shown an effect on TG reduction. Petersen et al. (2020) evaluated the inclusion of 2–6 g of spices in three types of meals, and their effect on postprandial lipemia. TG levels were lower following the meal with 2 g of spices vs. the no-spice meal (−18 ± 6 mg/dL (0.20 ± 0.06 mmol/L) (*p* = 0.015)) [82]. In concordance, a clinical trial showed the effectiveness of a nutritional supplement (mix spices: cuminum, piper nigrum, solanum nigrum) in addition to the standard statin therapy, in TG levels, after supplement intake together with standard therapy, and showed significant decrease in TG (338.2 ± 256.1 mg/dL (3.82 ± 2.89 mmol/L) to 216.9 ± 102.6 mg/dL (2.45 ± 1.15 mmol/L)) [83].

Regular consumption of fruits has been linked to cardiometabolic health due to their high content of bioactive compounds [84]. Some clinical trials have investigated the relationship between fruit consumption and TG levels. A randomized crossover intervention study conducted in 2020 determined that two whole apple (WA) consumption decreased serum TG (WA: 1.17 mmol/L; CB: 1.30 mmol/L; *p* = 0.021) [84]. On the other hand, incorporating 280 g of frozen raspberry in the usual diet decreased TG levels, but without statistical significance (−0.17 ± 0.68, *p* = 0.48). Although there were no significant differences in TG, there were significant differences in three species of secondary metabolites: TG (16:1/32:1), TG (18:2/32:2) and TG (18:0/32:0) decreased after the raspberry intervention (respectively, −0.96 ± 1.83, −1.04 ± 1.99, and −0.93 ± 2.17 μmol/L; *p* = 0.02, 0.02, and 0.04) [85].

One study showed that regular consumption of green/roasted coffee blend (three servings/day of the blend) decreased serum TG levels in hypercholesterolemic subjects (103.3 ± 7.5 mg/dL (1.16 ± 0.08 mmol/L) vs. 82.9 ± 6.1 mg/dL (0.93 ± 0.06 mmol/L) (*p* = 0.027)) [86].

There is variability in the strategies proposed to reduce TG, however, it can be concluded that consuming fruits regularly, including spices in meals, such as cuminum, piper nigrum, and solanum nigrum, and incorporating foods high in bioactive compounds, such as green tea, could be a strategy for cardiovascular health care, related to TG reduction. However, there is a lack of scientific evidence to clarify the precise recommendation to include these foods in the diet and obtain benefits over TG (Table 4).

## 5. Nutrients and Bioactive Compounds

### 5.1. Omega-3

The treatments to reduce TG are fibrates, niacin, statins, and omega-3. Fibrates and niacin have tolerability problems, such as flushing, liver-toxic effects, and myopathy. Statins are a drug that helps to reduce LDL cholesterol levels in patients with dyslipidemia, but their effect is not effective in controlling TG levels. Conversely, omega-3 has proven the effect with good tolerability; reports support that it has a safety and tolerability similar to that of placebo [87,88]. The recommendations of the American Heart Association (AHA) in patients with HTG are 2–4 g/day of eicosapentaenoic acid (EPA) + docosahexaenoic acid (DHA) [89].

Omega-3 is a PUFA and an essential fatty acid that comes in several forms [90]. EPA and DHA are the most investigated to protect against CVD [91], and its dietary sources are oily fish, fish oil, and particular types of seafood [92]. 

Several studies have demonstrated the triglyceride-lowering effect of omega-3 in patients with alterations in serum lipids (Table 5) [89,92,93,94]. Omega-3 supplementation of 2 and 4 g/day from 8 weeks to 38 months showed a significant reduction in serum TG concentrations compared to the placebo group (*p* = 0.017) that received olive oil or corn oil [89,93,95]. A study compared the doses of 2 and 4 g/day, finding significant changes in fasting and postprandial TG only in the 4 g group after 8 weeks (both *p* < 0.001) [94].

The ALA can be converted into EPA and DHA, although the effectiveness of this process within the human body is controversial. It is indeed a fatty acid with greater availability and affordability in sources of plant origin in foods such as flaxseed, walnut, soybean, pumpkin seeds, canola, and olive oil [92], and for this reason, Zhou et al. compared the effects of ALA with those of EPA and DHA on lipids and inflammation status over 12 weeks in patients with hypercholesterolemia. After the intervention, the groups that received 1.8 and 3.6 g/day of EPA + DHA significantly decreased TG levels, unlike the groups that took 4.2 and 7.2 g/day of ALA, where no changes were observed.

Omega-6 is another essential fatty acid and this lipid possibly increases the risk of chronic diseases since a diet rich in this nutrient promotes pro-thrombogenic, pro-aggregating and vasoconstriction effects. For these reasons, the omega-6/omega-3 ratio has the potential to play a role in the prevention of these diseases. Lee et al. investigated the impact of a diet with a low omega-6/omega-3 in a randomized controlled crossover trial. After a dietary intervention period in obese and cardiometabolically ill subjects, TG concentrations decreased from baseline (*p* < 0.01) and the proportion of subjects meeting criteria for HTG decreased from 34.4% to 20.3% (*p* = 0.020). However, the authors caution that the results of this study should be interpreted with care because the diet was also high in protein and fat and low in carbohydrates [96].

In patients with T2DM, the supplementation with fish oil showed great results. In a randomized, double-blind, placebo-controlled trial with 4 g/day of fish oil for 6 months, the TG concentrations decreased considerably (−21.25%) and were better than the control group that received corn oil (2.89; *p* = 0.007) [97]. Tremblay et al. used 5 g/day in a crossover study for 8 weeks in each intervention, and fish oil reduced fasting TG concentrations by 9.7% compared to placebo [98].

Familial hypercholesterolemia (FH) is an inherited disorder and the most common monogenic cause of hypercholesterolemia. Patients with FH mainly have LDL receptor mutations that cause increased LDL cholesterol levels. FH has been studied in a crossover trial with standard care and a period of no treatment or omega-3 supplementation treatment of 4 g/day. The results showed a 20% reduction in plasma TG compared to standard care. The authors found that although patients with FH had normal fasting plasma TG levels, their postprandial TG response was impaired and after the omega-3 supplementation these abnormalities were partially corrected [99,100].

Icosapent ethyl is a safety pure ethyl ester of EPA approved by the Food and Drug Administration (FDA) and recommended by the ADA as an adjunct to diet to reduce TG and cardiovascular risk in adults [88,101,102]. In two placebo-controlled, randomized, double-blind trials in women with high TG levels, the supplementation with 4 g/day icosapent ethyl for 12 weeks improved the TG concentrations compared to placebo [103]. In another data analysis of one of these trials (ANCHOR Study), women with high TG levels and T2DM and statin-treated subjects with increased CVD risk had the same effect with the icosapent ethyl [88,101].

Icosapent ethyl is only available as a medication, unlike over-the-counter omega-3 supplements that are a mix of DHA and EPA. The downside of this supplement is that it can promote oxidative stress and increase LDL cholesterol levels in some patients [103,104]. To evaluate the effect on TG of these fatty acids, a comparative study of these fatty acids was carried out in cardiac surgery patients with HTG, and after 3 years of supplementation the combination of DHA and EPA decreased TG concentrations compared to the group of icosapent ethyl (*p* = 0.005) [105].

Patients with chronic kidney disease have an increased cardiovascular risk. Alterations in lipid metabolism result in dyslipidemia characterized by alterations in TG, TG-rich lipoproteins, and LDL cholesterol. Statins are recommended as first-line in this population; however, residual risk may still remain despite therapy. Given that this population is more likely to die from CVD than from disease progression, it is necessary to look for adjuvants that reduce cardiovascular risk. With this objective, a placebo-controlled, randomized, double-blind, clinical study was carried out in patients with chronic kidney disease and high TG levels, 4 g/day of icosapent ethyl for 12 weeks reduced 16.9% TG concentrations compared to placebo [102].

Most of the research has focused on omega-3 supplementation and not on consuming foods rich in this nutrient, such as fish. Controlling the amount of omega-3 that is consumed from fish in the diet is difficult to determine since the amount in fish species depends on the processing method and the environment [90]. In one study, the consumption of omega-3 from the consumption of trout or fish oil supplement was compared for 8 weeks in subjects with dyslipidemia, and although the TG decreased in the two groups, the effect was more pronounced in the group that consumed fish (*p* = 0.003). The authors mention that the difference in the effect can be driven by other compounds in the fish or due to the modification of the diet; therefore, the consumption of the supplement does not replace the consumption of fresh fish [91].

**Figure 1:**
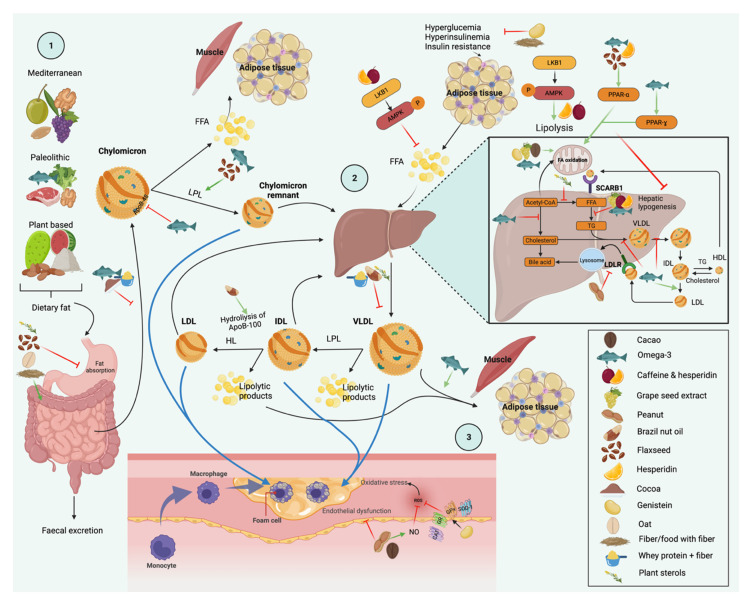
Modulation of lipid metabolism by food and bioactive compounds, adapted from [9,16,18,62,64,66,68,90,92,96,97,98,100,106,108,109,110,111,112,113,114,115,116,117]. **1.** The composition of diet, macronutrients, and adherence to dietary patterns may have an impact on lipid profile [16,17,18]. Dietary fat after a meal is hydrolyzed within the intestine. Food and its components can modulate the synthesis or release of fatty acids through different mechanisms at the intestinal, hepatic, muscular, and adipose tissue levels. Dietary fiber present in oats, psyllium, and flaxseed improve TG by the reduction of fat [64,108] and carbohydrate absorption [62], increasing fecal lipid excretion by the intestine [64] and the effect on gastric emptying [108]. For its part, cocoa could reduce degradation (release) of fatty acids from the fat in food [111], while whey protein low in fiber could decrease the synthesis of chylomicrons by enterocytes [115]. Flaxseed also increases LPL concentrations and the subsequent hydrolysis of TG rich lipoproteins [64]. Omega-3 mechanisms involve the downregulation of monoacylglycerol and diacylglycerol acyltransferase activities, expression of apoB-48 mRNA, and an increase in post-translational degradation of newly synthesized ApoB-48 [100] and LPL activity [106]. Plant sterols interfere with the absorption of fatty acids within the intestinal lumen [106]. **2.** At the hepatic level, genistein and fiber could reduce the uptake of fatty acids from TG through improvements in blood glucose [9,114] and a reduction in their transformation into TG [114]. Brazil nut oil and whey protein could reduce hepatic VLDL synthesis [68,115]. Furthermore, brazil oil and whey protein decrease TG due to the hydrolysis of apo-B100 [68] and VLDL remnant synthesis [115], respectively. Flavonoids from cacao, genistein, and grape seed inhibit fatty acid synthase and hepatic lipogenesis [112,113], as well as the increase of fatty acid oxidation [9,109,110,113] preventing their subsequent accumulation. Cacao modulates lipid metabolism through the regulation of gene expression by the activation of LKB1 and AMPK activation by phosphorylation. AMPK regulates lipid metabolism in liver, muscle, and adipose tissue and stimulates the oxidation of free fatty acids and ATP production [110]. Flaxseed and hesperidin activate the expression of genes encoding PPAR, which stimulate β-oxidation and the inhibition of fatty acid synthesis [66]. Omega-3 reduces TG synthesis [90], VLDL production and secretion [97,98], and the expression and maturation of the SREBP-1c, and increases the transcription of genes involved in the β-oxidation pathway by binding to PPARα and PPARγ [92], the rate of conversion of VLDL to LDL [98], β-oxidation of fatty acids [90], TG clearance with increasing of plasma lipolytic activity [97], and the removal of TG from chylomicrons [96]. Plant sterols modulate de novo hepatic lipogenesis and reduce circulating medium and large VLDL particles [106]. **3.** Peanut and cacao consumption could protect against endothelial and oxidative damage [62] besides the stimulation of nitric oxide production [62,110]. Genistein also exerts cardioprotective effects through inhibition of reactive oxygen species by increasing the expression of antioxidant gene enzymes [9]. All of these mechanisms could reduce the production of lipolytic products and future accumulation in the arterial wall, endothelial damage, and the development of CVD. Abbreviations: TG, triglycerides; VLDL, very low-density lipoprotein; LDL, low-density lipoprotein; IDL, intermediate-density lipoprotein; LPL, lipoprotein lipase; LDLR, low-density lipoprotein receptor; NO, nitric oxide; ROS, reactive oxidative species; LKB1, liver kinase B1; AMPK, adenosine monophosphate activated protein kinase; PPAR, peroxisome proliferator-activated receptors; SCARB1, scavenger receptor class B member 1; HL, hepatic lipase; FA, fatty acids; FFA, free fatty acids; SOD-1, superoxide dismutase 1; GPx, glutathione peroxidase; GR, glutathione reductase; CAT, catalase; SREBP-1c: sterol regulatory element-binding proteins-1c; CVD, cardiovascular disease.

The mechanisms of action of omega-3 in TG involve downregulation of monoacylglycerol and diacylglycerol acyltransferase activities, ApoB-48 mRNA expression, TG synthesis, VLDL production and secretion, expression and maturation of the sterol regulatory element-binding proteins-1c (SREBP-1c), as well as an increase in post-translational degradation of newly synthesized ApoB-48, plasma LPL activity, transcription of genes involved in the β oxidation pathway by binding to PPARα and PPARγ, conversion of VLDL to LDL, mitochondrial beta-oxidation of fatty acids, TG clearance with increasing of plasma lipolytic activity, and the removal of TG from chylomicrons (Figure 1) [92,96,97,98].

Other nutrients such as coenzyme Q10 (CoQ10) and plant sterols are important in cardiovascular risk. CoQ10 is a fat-soluble antioxidant endogenously synthesized in the body, and exogenous sources include pork, offal, fish, and vegetables. Reduced concentrations of CoQ10 have been detected in patients with CVD. To determine the possible complementary effects, Tóth et al. used omega-3 and coenzyme in subjects with dyslipidemia, and TG was reduced (*p* = 0.01) compared to the control group. The authors assume that joint administration increases the effect of each [90].

Plant sterols are bioactive compounds that among the derived plant stanols are called phytosterols. We obtain plant sterols with the diet in foods of plant origin, and a consumption of less than 1.5 g/day of plant sterols improves TG and LDL cholesterol levels [106,107]. In a clinical trial in subjects with high LDL cholesterol and TG, supplementation with plant sterols, EPA, and DHA for 4 weeks decreased TG compared to the control group (*p* < 0.001). Plant sterols can decrease TG concentration through the interference of the absorption of fatty acids in the intestinal lumen, reducing the circulating medium and large VLDL particles, as well as modulating hepatic de novo lipogenesis (Figure 1) [106].

Omega-3 has been shown to potentiate the effect of drugs [87,104,118,119]. To enhance the impact of statins in the controlling of TG levels, two studies supplemented diets with omega-3 4 g/day or placebo in subjects with HTG and mixed dyslipidemia for 8 weeks; the statin-plus-omega-3 group significantly decreased TG concentrations compared to the placebo group [87,118]. As we mentioned earlier, fibrates are part of the treatment to reduce TG levels. Kon et al. used this drug plus omega-3s in HTG patients in a parallel, randomized, single-blind, placebo-controlled study. The group with fenofibrate plus omega-3 reduced TG levels better compared to the group that received only fenofibrate (*p* = 0.001).

Previous studies have reported that the combination of omega-3 and a cyclooxygenase inhibitor improves the effect of reducing lipid concentrations and inflammation in mice. To investigate this effect in humans, Saraswathi et al. treated subjects with obesity and dyslipidemia with naproxen and omega-3 for 12 weeks in a prospective, open-label, randomized study. Although treatment with omega-3 + naproxen and omega-3 alone showed a trend towards a reduction in TG concentrations, the difference with the control group was not significant [104].

Omega-3 is a safe nutrient with considerable evidence in favor of its effect on reducing TG concentrations in patients with metabolic disorders and diseases. Most of the interventions have been carried out with supplements or medications, and although we only have one study that involves the consumption of fish, it highlights the importance of the consumption of nutrients from food sources. Omega-3 can exert its effect synergistically or potentiating when administered with other types of nutrients such as coenzyme Q10 and plant sterols, in addition to co-administration with medications (Table 5).

**Table 5:** Effect of omega-3 on serum triglycerides.

Author, Year	Dietary Component	Dose/Time	Study Design n	Main results on Triglyceride Levels
Chan, 2016 [99]	Omega-3 + standard care Standard care (control group)	Omega-3 4 g/day (46% EPA and 38% DHA in ethyl ester form) 8 weeks intervention-8 weeks washout-8 weeks intervention	Open-label, randomized, crossover intervention trial 20 subjects with FH (18–70 years)	↓ TG with omega-3 + standard care 113–75 ± 12.25 mg/dL ^a^ 1.30 ± 0.14 mmol/L vs. 91.88 ± 7.88 (1.05 ± 0.09); *p* = 0.011 (−20%) compared with the control group.
Chan, 2016 [100]	Omega-3 + standard care Standard care (control group)	Omega-3 4 g/day (46% EPA and 38% DHA in ethyl ester form) 8 weeks intervention, 8 weeks washout, 8 weeks intervention	Open-label, randomized, crossover intervention trial 20 subjects with FH (18–70 years)	↓ postprandial TG with omega-3 supplementation total AUCs (−19% ^b^; *p* < 0.01) and incremental AUCs (−18%; *p* < 0.05).
Koh, 2016 [119]	Fenofibrate + omega-3 Fenofibrate Placebo	Fenofibrate 160 mg + 2 g omega-3 Fenofibrate 160 mg 2 months	Randomized, single-blind, placebo-controlled, parallel study 146 subjects with HTG (54 ± 1 y ^c^)	↓ TG placebo group (−7% ^b^; *p* = 0.05), fenofibrate group (−30% ^b^; *p* = 0.05) and fenofibrate plus omega-3 group (−41% ^b^; *p* = 0.05). ↓ TG fenofibrate plus omega-3 group compared with placebo and fenofibrate (*p* = 0.001).
Mosca, 2016 [103]	Icosapent ethyl Placebo	4 g/day 12 weeks	Data of two placebo-controlled, randomized, double-blind trials (MARINE and ANCHOR) 215 womens with elevated and high TG levels (49–73 years)	MARINE ↓ TG with icosapent ethyl 4 g/day −27% (39) ^d^ compared to placebo −8.6% (54) (−23%; *p* = 0.0327) ANCHOR ↓ TAG with icosapent ethyl 4 g/day −20% (25) ^d^ compared to placebo 4.5 (40) (−22%; *p* < 0.0001).
Tremblay, 2016 [98]	Omega-3 Corn and soybean oil (placebo group)	5 g/day of fish oil (3 g/day of EPA (64%) and DHA (36%)) 5 g/day 8 weeks intervention, 12 weeks wash-out period, 8 weeks intervention	Double-blind, randomized, placebo-controlled, crossover study 10 mens with T2DM (54.7 ± 7.6 y ^c^)	↓ fasting TG omega-3 compared with the placebo (−9.7% ^b^; *p* = 0.05).
Su, 2017 [89]	Omega-3 Omega-3 Olive oil (placebo group)	2 g/day (920 mg EPA and 760 mg DHA) 4 g/day (1840 mg EPA and 1520 mg DHA) 8 weeks	Multicenter, randomized, double-blind, placebo-controlled, parallel study 251 subjects with HTG (20–79 years)	↓ TG in the 4 g/day (−32.1%(−38.0–−25.6%)) ^e^ and 2 g/day (−29.7% (−35.4–−23.4%)) ^e^ groups was larger than in the placebo group (−5.4% (−14.8–5.1%)) ^e^ (*p* < 0.001).
Tóth, 2017 [90]	Omega-3 Omega-3 + CoQ10 Unaltered statin therapy (control group)	2.52 g/day 2.52 g/day omega-3 + 200 mg/day CoQ10 3 months (±1 week)	Randomized double-blind trial with a statin treatment parallel group 105 subjects with dyslipidemia and elevated TG (57.24 ± 11.77 y ^c^)	↓ TG in omega-3 group to 143.5 ± 70.88 mg/dL (1.64 ± 0.81 mmol/L ^c^; *p* = 0.004) and omega-3 + CoQ10 to 124.25 ± 56.88 (1.42 ± 0.65 ^c^; *p* = 0.01) in comparison to the control group 221.37 ± 193.38 (2.53 ± 2.21) ^c^
Wang, 2017 [97]	Omega-3 Corn oil (placebo group)	4 g/day of fish oil (1.34 g EPA and 1.07 g DHA) 6 months	Randomized, double-blind, placebo-controlled trial 100 subjects with T2DM and abdominal obesity (65.4 ± 5.3 y ^c^)	↓ TG from baseline to 6 months in the omega-3 group 140 ± 80.5 mg/dL ^c^ (1.60 ± 0.92 mmol/L) to 151.38 ± 78.75 (1.73 ± 0.90); (*p* = 0.006). ↓ TG in the omega-3 group compared with the placebo group after 6 months (−21.25% ^b^ vs. 2.89% *p* = 0.007).
Zibaeenezhad, 2017 [91]	Omega-3 from fish oil Trout fish twice weekly	2 g/day (180 mg EPA and 120 mg DHA) Mean of 250 g with 1.4 of omega-3 (280 mg EPA and 160 mg DHA) per 100 g 8 weeks	Open-labeled, randomized trial 93 subjects with hyperlipidemia	↓ TG significantly in both groups, dietary-fish intake had a more pronounced effect than omega-3 supplementation (−30.75 ^b^ vs. −85.04; *p* = 0.003).
Brinton, 2018 [101]	Icosapent ethyl Placebo	4 g/day 12 weeks	Phase 3, multicenter, placebo-controlled, randomized, double-blind clinical study 146 women with increased CVD risk, TG 200–499 mg/dL and type 2 diabetes (>18 years)	↓ TG 4 g/day icosapent ethyl −17.4% (33.6) ^d^ compared to placebo 5.0 (40.5) (−21.5; *p* < 0.0001).
Kim, 2018 [87]	Rosuvastatin calcium + omega-3 Rosuvastatin + placebo	4 capsules rosuvastatin calcium 5 mg and 1 g/day omega-3 (380 mg DHA + 460 mg EPA) plus 1 tablet of placebo of rosuvastatin 20 mg/day 20 mg/day of rosuvastatin and 4 capsules of placebo 8 weeks	Multicenter, randomized, double-blind, placebo-controlled study 201 subjects with residual HTG (19 to 80 years)	↓ TG in the rosuvastatin + omega-3 group compared with the rosuvastatin + placebo group (−26.3% ^b^ vs. −11.4%; *p* < 0.001).
Stroes, 2018 [93]	Omega-3 Olive oil (placebo group)	2 g/day 2 g/day 12 weeks	Randomized, multicenter, double-blind, parallel-group trial 156 subjects with severe HTG (≥18 years)	↓ TG from baseline to the week 12 endpoint between 2 g omega-3 treatment when compared with the placebo group, −14.2% ^b^ (−26.2%, −2.8% ^d^); *p* = 0.017.
Blom, 2019 [106]	Low-fat spread with plant sterols and (EPA) + (DHA) from fish oil Low-fat spread with sunflower oil and no added plant sterols (placebo group)	2.0 g/day plant sterols and 1.0 g/day EPA + DHA 4 weeks	Randomized, double-blind, placebo-controlled, parallel group 259 subjects with elevated LDL cholesterol and borderline-high to high TG concentrations (18–75 years)	↓ TG in the treatment group compared with the placebo group (− 16.0% ^b^ to − 4.9); *p* < 0.001.
Miller, 2019 [88]	Icosapent ethyl Icosapent ethyl Placebo	2 g/day 4 g/day 12 weeks	Phase 3, multicenter, placebo-controlled, randomized, double-blind clinical study 246 statin-treated subjects at increased cardiovascular risk with TG 200–499 mg/dL (>18 years)	↓ TG 4 g/day icosapent ethyl group –16.6% ^b^ (32.3) ^d^ compared to placebo group 5 (44.4) (−19.9%; *p* < 0.0001).
Sezai, 2019 [105]	Icosapent ethyl EPA + DHA	1.8 g/day 0.93 g/day EPA + 0.75 g/day DHA 3 years	Single-blind study 87 cardiac surgery patients with HTG (≥20 to <90 years)	↓ TG in both groups from baseline to the 6 months, 1, 2 and 3 years; *p* < 0.05 ↓ TG after 2 (*p* = 0.032) and 3 (*p* =0.005) years in the EPA + DHA group compared with the EPA group.
Vijayaraghavan, 2019 [102]	Icosapent ethyl Icosapent ethyl Placebo	2 g/day 4 g/day 12 weeks	Phase 3, multicenter, placebo-controlled, randomized, double-blind, clinical study 83 subjects with TG 200–499 mg/dL and chronic kidney disease in stage 3 (>18 years)	↓ TG 4 g/day icosapent ethyl group –17.1% ^b^ (43.2) ^d^ compared to placebo group 5.9 (36.0) ^d^ (−16.9%; *p* = 0.0074).
Zhou, 2019 [92]	EPA + DHA from fish oil EPA + DHA from fish oil ALA from flaxseed oil ALA from flaxseed oil Corn oil (placebo group)	1.8 g/day 3.6 g/day 4.2 g/day 7.2 g/day 12 weeks	RCT, double-blind trial 123 subjects with moderate to high hypercholesterolemia (40–65 years)	↓ TG in 1.8 g EPA + DHA group (−11.99% ^b^; *p* = 0.008) and 3.6 g EPA + DHA (−15.78; *p* = 0.024). The changes in those two groups differed significantly compared with the placebo group; *p* < 0.05.
Jun, 2020 [118]	Atorvastatin calcium + omega-3 Atorvastatin + placebo (placebo group)	4 capsules with atorvastatin calcium 5 mg and 1 g/day omega-3 plus 1 tablet of placebo of atorvastatin 20 mg/day 20 mg/day of atorvastatin and 4 capsules of placebo (olive oil 1000 mg) 8 weeks	Randomized, double-blind, placebo-controlled, parallel-group, and phase III multicenter study 200 subjects with mixed dyslipidemia (20–79 years)	↓ TG in the omega-3 plus atorvastatin group after intervention (−29.8 ± 3.2 ^a^; *p* < 0.001). ↓ TG in the omega-3 plus atorvastatin group than in the placebo group (−29.8 ± 3.2% ^a^ vs. 3.6 ± 3.7); *p* < 0.001.
Nicholls, 2020 [95]	Omega-3 Corn oil (placebo group)	4 g/day (EPA + DHA) 4 g/day Median of 38.2 months	Double-blind, randomized, multicenter trial 13,078 subjects with high cardiovascular risk, HTG, and low levels of HDL cholesterol (62.5 years ^c^)	↓ TG in the omega-3 treatment group compared with the placebo group (−19.0% ^b^ vs. −0.9; *p* < 0 .001).
Saraswathi, 2020 [104]	Omega-3 Naproxen + Omeprazole Omega-3 + Naproxen + Omeprazole Standard nutrition counseling (control group)	4 g/day (300 mg EPA and 200 mg of DHA per gram) 440 mg/day Naproxen + omeprazole 20 mg/day 4 g/day omega-3 (300 mg EPA and 200 mg of DHA per gram) + 440 mg/day naproxen + omeprazole 20 mg/day 12 weeks	Randomized prospective open-label study 34 obese dyslipidemic (39–58 years)	↔ absolute fold change TG in omega-3 (−57 ± 24) ^a^ and omega-3 + naproxen-treated subjects (−63 ± 23) ^a^ compared with control group (−6 ± 26) ^a^
Teramoto, 2020 [94]	Omega-3 Omega-3	2 g/day 4 g/day 8 weeks	Multicenter, open-label, randomized study 37 subjects with HTG (≥ 20 years)	↓ fasting TG over the 8-week study period from baseline 194.4 ± 48.6 mg/dL ^c^ (2.2 ± 0.55 mmol/L) to 144.9 ± 43.1 (1.65 ± 0.49); *p* < 0.001 and mean 4 h postprandial TG 278.2 ± 70.5 mg/dL (3.17 ± 0.8 mmol/L) to 202.3 ± 78.3 (2.31 ± 0.89) only in the 4 g group (*p* < 0.001).
Lee, 2021 [96]	HMR Regular dietary pattern (control group)	Higher protein and fat content, lower carbohydrate content, and a lower omega-6/omega-3 ratio (<4) 4 weeks intervention, 2 weeks wash out period, 4 weeks intervention	Monocentric, controlled, randomized crossover trial 64 obese and cardiometabolically unhealthy subjects (≥ 40 years)	TG were reduced in the HMR group 134.1 ± 64.6 mg/dL ^c^ (1.53 ± 0.74 mmol/L) to 115.6 ± 48.4 (1.32 ± 0.55); *p* < 0.01.

EPA: eicosapentaenoic acid; DHA: docosahexaenoic acid; FH: familial hypercholesterolemia; TG: triglycerides; AUC: area under curve; HTG: hypertriglyceridemia; T2DM: type 2 diabetes mellitus; CoQ10: coenzyme Q10; CVD: cardiovascular disease; LDL: low density lipoprotein; ALA: α-linolenic acid; HMR: home meal replacement; g/day: grams per day; mg: milligrams; mmol: millimoles; L: liter; dL: deciliter; ↓ decrease; ↑ increase; ↔ No significant difference; ^a^ Values are reported on mean and SEM. ^b^ Values are % of change. ^c^ Values are reported on mean and standard deviation. ^d^ Values are change from baseline, % median and interquartile range. ^e^ Values are reported on means (95% CIs).

### 5.2. Flavonoids

There is increasing evidence of the effects of dietary flavonoid consumption on cardiovascular health [109,120]. Dietary flavonoids are a diverse group of polyphenolic compounds which are responsible for plant structure pigments [120,121]. They are classified in more than 5000 subclasses [122]. The most common subspecies are flavanols (epicatechin), flavones (luteolin), flavanones (herperidin), flavonols (quercetin), flavan-3-ols, proanthocyanidins (oligomeric and polymeric flavonoids), isoflavones (genistein), and anthocyanins (cyanidin) [121,122]. They are present in fruits, vegetables, grains [120], herbs (curcuma) [123], beverages [120], cocoa, and dark chocolate [109,110].

To date, different RCT have evaluated the efficacy of the consumption of different flavonoid subclasses from food or extracts such as curcuma [123], cocoa [110,111,124,125], dark chocolate [109], hesperidin [112,126], genistein [9], and grape seed extract [113] on different cardiovascular markers such as serum lipids. 

Reports have shown that consumption of cocoa and dark chocolate could modulate cardiovascular health [109] through improvements in lipid levels (decrease TG and increase HDL-C) [124,125]. These effects are mainly related to flavanols [124]. An RCT published in 2016 reports a significant reduction in TG with different doses ranging from 500 to 1.5 g of curcuma tea. However, a higher dose of curcuma tea consumption (1.5 g) led to greater improvements in blood TG levels with a decrease of 12.5% (*p* < 0.05) compared with baseline values in hypercholesterolemic individuals after 2 months [123]. In addition, a basal and postprandial effect of flavonol-rich cocoa on cardiometabolic parameters has been investigated in several studies [110,111,124,125]. Gutiérrez and colleagues evaluated the effect of 25 mg of epicatechin from cacao (flavanol) in thirty hypertriglyceridemic subjects during 4 weeks. They demonstrated a significant reduction of 26.5% in TG in the epicatechin group compared with baseline values (26.5%, *p* < 0.05) [110]. The results are contradictory to those reported by other authors [111,124,125]. A randomized, placebo-controlled, double-blind crossover involving 48 adults found that the intake of the same dose of epicatechin (25 mg) for 2 weeks does not reduce TG in overweight, obese, and MS adults. The author supported the idea that the duration of 2 weeks was too short to show improvements in the cardiometabolic parameter and lipids profile [111]. Consistent with this finding, two additional studies have shown that 2.5 g/day of capsules of flavanol-rich cocoa cannot reduce serum TG concentration after 2 and 12 weeks, respectively [124,125]. There was no difference on lipid profile between the groups at baseline [124] and postprandial TG in hypertensive and obese populations [125]. Notably, an RCT study involving 84 young patients with obesity, dyslipidemia, and T2DM assigned to receive 2 g/day of dark chocolate with high content of flavonoids found that TG decreased 32.8% after 6 months compared with day 0 (*p* < 0.05) [109]. 

Other studies have also demonstrated the hypotriglyceridemic effects of flavanones and isoflavones in cardiometabolic risk individuals. Daily consumption of 500 mg of hesperidin with or without caffeine had no significant effect on serum TG levels in subjects with moderately high levels of TG. However, despite not showing statistical significance, there is a trend of a 29.33 mg/dL (0.33 mmol/L) reduction in TG levels after 8 weeks with an intervention with hesperidin over 75 mg of caffeine. According to the authors, these results may be due to previous reports where TG in obese people do not improve in response to overproduction or insulin resistance [112]. Years later, Yari et al. in an RCT evaluated the same dose of hesperidin (500 mg/day) in 49 MS patients showing a greater improvement on TG in the IG compared with placebo (mean difference (−49.09 mg/dL (−0.56 mmol/L) vs. −8.93 (−0.1); *p* < 0.05) [126]. Furthermore, the supplementation of high genistein, isoflavones in capsules (54 mg/day) demonstrated a mean difference of −35.28 mg/dL (0.4 mmol/L) (95% CI of −61.35 to −9.21) (CI −0.7, 0.1); *p* = 0.01) after 12 weeks compared with baselines in a postmenopausal woman with T2DM. According to these findings, the reduction in TG after genistein supplementation could be explained by improvements in blood glucose, through the selective binding of genistein to estrogen receptors, which modulate glucose and lipid metabolism. Other cardioprotective and lipid-metabolism-modulating effects of genistein are related to its ability to inhibit reactive oxygen species through the expression of antioxidant enzyme genes such as superoxide dismutase, glutathione peroxidase, glutathione reductase and catalase, and the reduction of inflammatory cytokine production by inhibiting the NF-kB pathway [9]. In 2021, the effects of flavonoids in diet on serum TG were confirmed. A randomized controlled trial investigated the effect of an intervention with a restricted-calorie diet with 300 mg/day from grape seed extract rich in flavonoid in 40 obese or overweight subjects during 12 weeks, which showed a reduction in TG of −17% (*p* < 0.05) compared to initial values [113].

Together, these dates suggest that flavonoids might contribute to cardiovascular health by lowering TG levels and modulating lipid metabolism through the inhibition of fatty acid synthase and the subsequent hepatic lipogenesis [112,113], to the increase of fatty acid oxidation [9,109,110,113], and to the regulation of gene expression by the activation of skeletal muscle sirtuin 1, liver kinase B1 and 5′ adenosine monophosphate-activated protein kinase, and peroxisome proliferator-activated receptor gamma coactivator 1-a via their phosphorylation [110], and the expression of genes encoding the peroxisome proliferator-activated receptors (PPAR) [126]. The consumption of flavonoids from different food sources could improve TG concentrations from 10 to 30%. Further studies with a larger population are required to be able to define therapeutic doses of flavonoids in subjects with cardiovascular risk, as a consequence of the differences in bioavailability and bioactivity between subject [120]. However, the above gives us an approach to future dietary interventions for the population (Table 6).

### 5.3. Dietary Fiber

The positive effects of fiber intake on health have been demonstrated over time [127]. Dietary fiber is part of the plant-based foods [128] which are non-digestible and come from carbohydrates and lignin. Dietary fiber is commonly classified according to its solubility as soluble and insoluble [129]. This includes cellulose, non-starch polysaccharides, pectins, hydrocolloids, fructo-oligosaccharides, lignin, beta-glucans, and resistant starch [128,130]. The food groups with the highest fiber content are whole grain cereals, legumes [130], fruits, and vegetables [129]. Whole grains are the main source of insoluble fiber [128]; however, they are also composed of soluble fiber [129]. There is a lot of evidence about the health effects of dietary fiber consumption on metabolic health, insulin response, improvement in glucose, lipids, and the reduction in cardiovascular risk [128,129].

In this section, we present a summary of the main effects of dietary fiber from whole grains principally in the reduction of cardiovascular risk, through the improvement of blood lipid concentration (TG).

Several clinical studies have assessed the benefits of dietary-fiber-enriched diets in humans [115,131,132,133,134,135]. A clinical intervention with a low-fat, high-fiber diet (23–35 g/day) in obese and dyslipidemia adults has reported an increase by 2.8% in TG compared to starch-rich diets (without statistical significance). Authors have concluded the need to detail the information of fiber fractions in the foods included in the diet and to target interventions with different fiber composition [134]. In this sense, other studies have focused on evaluating diets according to their glycemic index and the type of fiber in food and/or fiber-rich cereals. RCTs that have specifically evaluated the type of carbohydrate have shown a reduction in TG [114,131,132,133]. According to a study carried out with 298 T2DM participants who were randomized into a whole grain oat group with two doses of oat (50 or 100 g) or a usual care group (healthy diet) during 30 days, a little reduction in TG was observed with both amounts of oats. Nevertheless, the consumption of 50 g of oats caused a significant reduction in TG after intervention compared to baseline (−7.88 mg/dL (−0.09 mmol/L); *p* = 0.003). The authors suggest a lipid lowering through a reduction in the absorption of carbohydrates and lipids at the intestinal level. These properties are attributed to ß-glucans [131]. In another study with a longer intervention (3 months) MS subjects received a low-glycemic-index diet. A higher intake of whole grain cereal foods was related to a greater reduction in serum TG levels (−16.9 ± 2.8 mg/dL (−0.19 ± 0.03 mmol/L); *p* = 0.538) compared with refined cereal products group (*p* < 0.05) [132]. Tessari et al. [133], and Fatmah et al. [114] evaluated and compared the effect of 100 g of fiber-rich breads or white bread on lipid profile concentrations in adults and older adults with diabetes. Plasma TG levels were greater (*p* < 0.05) in the bread rich in beta glucans than the white bread after 6 months. It did not significantly change in either group (functional bread and white bread) between baseline and end values [133], while a fiber-rich biscuit consumption demonstrated a significant reduction in TG of 11.03 mg/dL (0.13 mmol/L) after 4 weeks compared with the initial evaluation (*p* < 0.05). In addition, the study showed a positive correlation between glycemic control and TG, which could hypothesize the reduction in blood glucose transformation into TG [114]. Research on soluble fiber carried out by Pal et al. [108] reported that a supplementation with 5 g/day of psyllium had improvements in TG: concentrations were significantly lower in the psyllium group after 6 months compared to baseline (−12.7%; *p* < 0.05). The improvement in TG suggests a possible effect of fiber on the gastric emptying and reduction in the absorption of lipids in the intestine [108]. Even so, in an intervention study, a supplementation with 30 g/day of gum arabic indicated a reduction in TG (119.88 ± 51.63 mg/dL (1.37 ± 0.59 mmol/L) vs. 134.75 ± 55.13 (1.54 ± 0.63); *p* = 0.17) after 3 months, but no significant differences were observed between groups and time. The above results may be due to the number of subjects analyzed and the time of the intervention [135]. On the other hand, an RCT in 41 dyslipidemia adults evaluated the effect of two diets of whey protein that are low in fiber (10 g/day) and high in fiber (30 g/day), respectively, on fasting TG. The author observed a greater reduction in TG at 12 weeks with a whey protein low-fiber intervention compared with baseline and other groups (*p* < 0.05). No significant difference was reported with the high-fiber protein compared with the groups. The possible mechanism by which TG are reduced may be due to decreased chylomicron synthesis by enterocytes and hepatic synthesis of VLDL and VLDL remnants [115]. Similar results were reported by Anggadiredja et al., in an RCT whose objective was to compare an intervention with a soluble-fiber–multivitamin combination with insoluble fiber in dyslipidemic adults. The combination of 4 g of soluble fiber with multivitamins during 6 weeks does not seem to have an effect on serum TG levels compared with baseline and control group [136].

Based on the available published RCT, low-glycemic-index diets, the substitution of fiber-rich breads in place of refined cereals, the addition of at least 50 g/day of whole grains such as oatmeal and an extra 5 g of psyllium supplementation per day, appear to have improvements in serum TG concentrations. However, the addition of fiber to commercial products such as protein or multivitamins has shown no difference in TG concentrations. Therefore, the recommendation to increase consumption of whole grains in the diet and the replacement of refined grain with this remains an ideal strategy in people with metabolic risk factors. It is also part of one of the main evidence-based strategies of the dietary guidance of AHA 2021 to promote cardiovascular health [137].

Another component of the diet that has been studied is oats (*Avena sativa* L.). This food is rich in carbohydrates and fiber such as beta-glucans, a highly viscous soluble fiber that is found naturally in the cell wall. In addition to this, it contains vitamins, minerals, and antioxidants that improve the nutritional value of the diet [138,139,140].

A study found a significant decrease in TG concentrations in hypercholesterolemic patients after the intervention for 30 days with 40 g/day of oat bran and nutritional advice based on the ten steps for a healthy diet published by the Ministry of Health compared to the group that received starch corn and rice flour in the same dose as well as nutritional advice [138]. A possible mechanism proposed by several authors to explain this effect is the formation of a gelatinous layer in the intestinal lumen by beta-glucans that inhibit the absorption of fats and carbohydrates [138,139,140]. On the other hand, two studies did not see positive results in the same population: Gulati et al. used 70 g of oats for 4 weeks and Cicero et al. a formulation with 3 g/day of oat beta-glucans for 2 months [139]. 

Another type of fiber that is important to reduce the risk of CVD is fiber from cereals and resistant starch. However, a crossover clinical trial in 19 MS patients performed an intervention for 4 weeks with a diet with a mean dietary fiber content of 68 g/day (based on arabinoxylan and resistant starch) and 4 weeks with a Western-diet-style diet rich in refined carbohydrates with an average dietary fiber content of 21 g/day without observing significant changes in serum TG [141].

The inclusion of dietary fiber from oats in the diet can be a valid strategy to prevent the onset of CVD [140]. The consumption of refined starches and added sugars should be avoided as prevention and a good option to replace them is egg proteins and unsaturated fatty acids (Table 7).

## 6. Molecular Nutrition

The consequence of different dietary interventions over cardiovascular risk parameters such as TG levels and its association with gene variants, gene expression, or gene methylation status is assessed in the following projects as either primary or secondary outcomes. 

### 6.1. Genetic Variants

Uncoupling protein 3 (UCP3) is a mitochondrial transporter. Studies indicate its participation in energy regulation; a decrease in its expression or function could therefore increase the storage of adipose tissue and reduce energy expenditure. A genetic variant located in the promoter of the *UCP3* gene (–55C>T; rs1800849) has been associated with increased BMI in the C/C genotype. An RCT investigated the influence of this variant on a high-protein, low-carbohydrate, low-calorie dietary intervention or a standard protein, low-calorie diet in obese patients, and after 9 months there was a significant reduction in TG in patients with the C/C genotype with the high-protein, low-carbohydrate, low-calorie diet. The possible molecular mechanisms of this effect could be explained by the location of the genetic variant, which, being in the promoter, is related to mitochondrial oxidation and is able to influence the response to lipids; another possible explanation is that the distribution of nutrients from the diet interacts with the metabolic response and genetic variant [142].

Neuropeptide Y (NPY) is secreted in the arcuate nucleus and enhances appetite, so that increased signaling contributes to the development of cardiometabolic diseases. An association has been found between genetic variants of the *NPY* gene and the early onset of these diseases. The variant (–399G>A; rs16147) of the *NPY* gene is located in the promoter region of the gene and appears to explain almost half of the variation in gene expression levels. This genetic variant was studied in an RCT in obese patients where the intervention consisted of two hypocaloric diets low in carbohydrates or fats, and after 3 months of treatment no significant changes in TG were observed [143].

Apolipoprotein E (APOE) is a polymorphic protein involved in the uptake of lipoprotein particles by the liver, making it a target for preventing or treating atherosclerotic vascular disease. Its genetic variants result in 3 isoforms (E2, E3, and E4) that differ by single amino acid changes and alter their conformational structure [144,145]. The APOE4 genotype confers greater sensitivity to changes in fatty acids in the diet and it has been associated with an increased risk of mortality from coronary heart disease, while E2 markedly reduced its affinity for LDL receptors and may exert a greater influence on the modulation of serum TG. In an RCT conducted in subjects at risk of developing metabolic syndrome, a significant reduction in TG was found in E2 and E4 carriers when SFA was replaced with MUFA and low glycemic index carbohydrates compared to the E3/E3 genotype [145].

The circadian system is related to metabolism, since it influences the coordination of metabolic pathways through the modulation of the genes involved, so that alterations in the circadian system can lead to dyslipidemia. Melatonin is a hormone produced by the pineal gland that is involved in this system by controlling circadian rhythms. A genetic variant (C>G; rs10830963) has been described in the melatonin receptor 1B (*MTNR1B*) that is related to overweight and dyslipidemia, in addition to having an interaction with dietary fat and serum lipids. Therefore, the participation of this variant in the intervention of hypocaloric diets (high in MUFA or PUFA) in obese patients was investigated and after 3 months of intervention no changes were observed with any diet [146].

Cannabinoid receptor type 2 (CB2R) is the peripheral cannabinoid receptor that regulates the inflammatory response. It is a common genetic variant (G>A; rs3123554) of the *CB2R* gene, and studies have found that carriers of the A allele have less pronounced improvement in weight, fat mass, and biochemical parameters, such as lipid profile, than non-carriers of the A allele after a hypocaloric diet [147]. Two studies have been carried out exploring the effect of this variant after weight loss secondary to hypocaloric diets. Aller et al. designed an intervention high in MUFA or PUFA in obese patients for 3 months and found a reduction in TG in non-carriers of the A allele after a diet rich in PUFA fatty acids. On the other hand, Primo et al. conducted an RCT with a hyperproteic, low-carbohydrate, and hypocaloric diet or a standard hypocaloric, protein diet in obese patients, finding a decrease in TG in non-carriers of the A allele with the hyperproteic, low-carbohydrate, and low-calorie diet [148].

The *BDNF* gene encodes the brain-derived neurotrophic factor which influences food intake and body weight control. Animal models with *BDNF*-engineered disruption have shown an increase in food consumption, while studies in humans have seen differences in BMI among genotypes and *BDNF* gene variants. It is also argued that the *BDNF* gene plays a role in glucose metabolism in humans [149,150].

An RCT analyzed the effect of the rs10767664 *BDNF* gene variant on the intervention with two hypocaloric diets with different fat distributions (one rich in PUFA and the other rich in MUFA) among obese individuals. The results revealed that individuals carrying the wild type allele (A) had an improvement in total cholesterol and LDL cholesterol in both diets. The diet rich in MUFA additionally showed improvement in the Homeostatic Model Assessment of Insulin Resistance (HOMA-IR) and insulin, but TG showed no change [149].

In another approximation with the *BDNF* gene, 80 obese Caucasian patients were enrolled in a non-randomized intervention with a hypocaloric diet of 1520 calories per day for three months. The macronutrients were distributed as 52% carbohydrates, 25% lipids, and 23% of proteins. It analyzed the repercussions of the gene variant *BDNF* rs10767664 A>T on weight, cardiovascular risk factors, and serum adipokine levels after intervention. After intervention a significantly higher reduction in TG levels was found, accompanied by greater decreases in weight, BMI, fat mass, waist circumference, insulin, HOMA-IR, and leptin in AA genotype carriers compared to other genotypes. This intervention trial is one of the few to analyze the effect of the *BDNF* gene variant rs10767664 A>T since most of them have been performed in cross sectional designs. Nonetheless, the functional consequence of this variant on *BDNF* gene expression or circulating levels was not assessed in this study [150].

A multicenter RCT in the US carried out in glucose-intolerant individuals with a polygenic risk score for cardiovascular disease found that a lifestyle intervention for preventing DM2 during a year showed a better effect on BMI, fasting glucose, and TG than metformin treatment or placebo regardless of genetic risk score for coronary artery disease [151].

The *CLOCK* gene is involved in circadian rhythm regulation, and it has been associated with cardiovascular risk. The association of gene variant *CLOCK* rs4580704 C>G with T2DM and CVD incidence was assessed in 7098 PREDIMED participants in a randomized clinical trial with a median follow-up of 4.8 years. The subjects were randomized either to an intervention with a Mediterranean diet supplemented with EVOO or to a Mediterranean diet supplemented with mixed nuts or a low-fat control diet. At baseline the TG levels were higher in T2DM than non-T2DM participants (132.5 ± 69.6 mg/dL vs. 141.5 ± 79, respectively). On the other hand, TG levels were similar among genotypes CC, CG, and GG. However, there was a non-significant trend of lower TG values for GG carriers (137.5 ± 75.5 mg/dL, 137.6 ± 75.1 and 132.6 ± 68.6, respectively). In addition, carriers of GG genotype showed lower weight, BMI, and waist circumference than subjects with other genotypes. It was observed that T2DM carriers of the G allele showed a decreased risk of stroke compared to the CC genotype (HR:0.61, 95% CI 0.40–0.94, *p* = 0.024), and the Mediterranean diet increased the protective effects of the G allele against T2DM. The G allele was also associated with protection against stroke in T2DM subjects. The authors pointed out the need of a circadian rhythm evaluation in the study to give more information on the effect of the *CLOCK* studied variant with chrono-disruption and its metabolic alterations [152].

A key piece to carry out precision nutrition is knowing gene-environment interactions. Evidence indicates that the response to dietary intervention with modifications in their macronutrients and energy on TG concentrations may be conditioned by genetic variants of the *UCP3*, *APOE*, *CB2R*, *CLOCK*, and *BDNF*, which emphasizes the importance of nutritional genetics.

### 6.2. Gene Expression

Arpón A and colleagues evaluated the effect on DNA methylation of genes related to intermediate metabolism, diabetes, inflammation, and the signal transduction process such as CPT1B and GNASAS, by two different interventions based on the Mediterranean diet plus olive oil or mixed nuts during five years. They found an association between PUFA content and methylation in the *CPT1B* gene (cg01081346 CpG), suggesting that some part of the preventive actions of the Mediterranean diet for T2DM and CVD could be mediated by methylation. Additionally, the subjects presented a reduction in LDL cholesterol and hypercholesterolemia and an increment of HDL cholesterol in both diets without changes in TG levels [153].

In the study conducted by Franck et al., blood gene expression was assessed as secondary analyses trough an 8-week supplementation with 280 g/day of frozen raspberries in subjects with abdominal obesity or overweight and slight hyperinsulinemia or HTG (see Section 4.3 for further information about trial). It showed a differential expression in 119 genes with a fold change greater than 1.25 but without significance after multiple testing corrections (FDR *p* > 0.05). After relaxing the criteria *p* < 0.001, 9 genes were up-regulated and 34 down-regulated post intervention [16].

A pathway enrichment analysis was performed to obtain a mechanistic perception of the genes differentially expressed. In this sense, most of the genes were pulled together into 12 intracellular transduction pathways associated with IL-1b, IL-6, and also Ras, Rho, and MAPK transduction pathways [85].

The modulation of gene expression by diet or foods has been shown in trials with the Mediterranean diet and raspberries, respectively. In both cases, there was an impact over methylation or expression. However, TG levels had no difference after interventions, which raises the perspective to give more attention when conducting nutritional interventions to modify gene expression in order to have health improvements in the long run (Table 8).

## 7. Conclusions and Future Perspectives

CVD persists as the leading cause of mortality in Western countries. Approximately 25–50% of the population has elevated TG (≥150 mg/dL), putting them at an increased level of highly atherogenic remnant-like particles, non-alcoholic fatty liver disease, and pancreatitis. Therefore, elevated TG are independently associated with increased atherosclerotic cardiovascular disease risk.

The importance of lowering plasma levels of TG has been called into question many times, but currently it is considered an integral part of residual cardiovascular risk reduction strategies, considering that high TG concentrations have been recognized as a risk factor and predictor of cardiovascular diseases comparable to LDL cholesterol [154]. Lifestyle changes, specifically dietetic strategies, are effective TG-lowering measures. The evidence between the effect of diet and components on HTG has been increased in the last years. In this regard, this review compiles exhaustively the most current and relevant clinical trials. Previous reviews have addressed the nutritional approach to HTG in conjunction with other cardiovascular risk factors, metabolic syndrome components, or other pathologies. This has resulted in limited depth and focus to the specific nutritional treatment of HTG.

Based on the dietary guidance to improve cardiovascular health of the AHA (2021) [137], we selected key words (healthy diet, food, nutrient, or bioactive component related to HTG decrease), to establish a search strategy in the PubMed database. We focused on nutritional strategies that had a positive impact in treating HTG. For this reason, sweetened or alcoholic beverages were not included in this review. Since the adult population with the presence of some metabolic alterations is the most affected by HTG, we decided to select RCTs with these subjects.

The most relevant findings of this review focus on macronutrient distribution changes such as fat or protein, low-carbohydrate diets, and caloric restriction. These strategies seem to be effective in reducing TG levels, as long as the food quality is prioritized. The Mediterranean diet was the dietary pattern with the most consistent evidence against HTG. Therefore, in this review the accumulated evidence for the effect of diet on HTG was 26 RCTs.

The use of omega-3 supplements was the dietary component strategy with the highest number of RCTs (n = 23) carried out with effective results to reduce TG, while intake of nuts and flaxseed was the food group most tested for lowering TG (n = 15). The inclusion of flavonoids or fiber on the diet had approximately ten RCTs (each one), testing their reduction effect on TG. In consideration of other foods such as soy, olive oil, low-fat milk, spices, fresh and dried fruits, and green coffee blend, the evidence was not sufficient to support their use on specific diets in subjects with metabolically altered states. In consequence, more studies are needed replicating the results that TG decrease after consumption of the previous recommendations.

From a genetics point of view, there is a lack of studies conducted to investigate the consequence of different interventions and considering genetic variants involved in TG metabolism. In the case of the included studies, there exist different analyzed genetic markers in genes such as *UCP3*, *NPY*, *APOE*, *MTNR1B*, *CB2R*, *CLOCK*, *BDNF*, and *CPT1B*; nonetheless, the functional consequence of the studied variants is not clear in most of them, and some are based only on previous association studies. Moreover, the studied variants may have a general repercussion in metabolism, but they do not impact TG levels or completely explain the outcomes.

We recognize that there is a lot of evidence on gene expression through in vitro or in vivo experiments that shows interesting mechanisms: for example, the liupao tea extract, traditional kefir, and Tibet kefir improve TG in serum and liver through the modulation of LPL expression in rats [155,156,157]. On the other hand, green tea supplementation reduces the metabolic damage of obesity by the improvement of insulin sensitivity, TG concentration, and expression of PPARα, PPARγ, and their target genes such as LPL, GLUT4, and adiponectin in obese dogs [158]. However, they are beyond the scope of this review. In this sense, only two interventions addressed an expression analysis through the methylation of promoters or a genomics approach.

The mechanisms of action of all dietary strategies described in the RCTs cited in this review are summarized in Figure 1. It suggests that the intake of a healthy diet performance on TG through the action of its foods, nutrients, and bioactive components at various levels of metabolism: downregulation of monoacylglycerol and diacylglycerol acyltransferase activities, ApoB-48 mRNA expression, TG synthesis, VLDL production and secretion, expression and maturation of sterol regulatory element binding proteins-1c (SREBP-1c), as well as an increase in post-translational degradation of newly synthesized ApoB-48 and ApoB-100, plasma LPL activity, transcription of genes involved in the β-oxidation pathway by binding to PPARα and PPARγ, conversion of VLDL to LDL, mitochondrial β-oxidation of fatty acids, TG clearance with increasing plasma lipolytic activity and the removal of TG from chylomicrons, inhibition of fatty acid synthase and the subsequent hepatic lipogenesis, the increase of fatty acid oxidation and regulation of gene expression by the activation of skeletal muscle sirtuin 1, liver kinase B1 and 5′ adenosine monophosphate-activated protein kinase, and peroxisome proliferator-activated receptor gamma coactivator 1-a via their phosphorylation.

The future perspective that the results of this review suggest is increasing scientific evidence to reach precision nutrition for HTG management. In this direction we suggest:

- The execution of systematic reviews and meta-analyses for specific nutritional strategies with enough evidence to obtain precise recommendations for each one.
- To replicate the results of dietary intervention efficient to lower TG in several phenotypes.
- To design and perform RCT with the main objective of measuring the effectiveness in reducing TG with dietary interventions; many have measured it secondarily.
- Future RCT should be with a larger number of participants and long term (at least 6 months).
- New molecular nutrition studies should clarify the diet mechanism of action on HTG through gene level.
- Increase knowledge about food science focused on maintaining healthy TG.
- To encourage the evaluation of the effect of modifying eating behaviors (healthy and unhealthy) through several techniques to reduce TG.
- To conduct more studies where joint strategies with prior evidence are assessed to decrease TG, examples: diet + one or more supplements of bioactive compounds or functional food.
- The use of technology in new dietary interventions on HTG could improve adherence to treatment.
- A new review could describe in detail the impact of the consumption of ultra-processed products on TG. The guidelines recommend reducing them in general, but sometimes this is not feasible for the population. For this reason, a more precise recommendation is needed.
- We invite those interested in implementing new clinical trials to include postprandial TG measurements, in addition to fasting. Fasting TG have long been associated with CVD and other cardiometabolic conditions. Evidence suggests that non-fasting TG (i.e., measured within 8 h of eating) better predict CVD than fasting TG, which has led several organizations to recommend non-fasting lipid panels as the new clinical standard [159].

In conclusion, the consumption of omega-3 supplements and the Mediterranean Diet are the best documented nutritional interventions for the reduction of TG. Future nutritional approaches or the replication of the proposed approaches to treat HTG should be strengthened with more high-quality scientific evidence to approach precise recommendations. The adult population exhibits certain characteristics and needs within every decade of life that should guide in the generation of nutritional scientific evidence to find the best approach to healthy eating behaviors to prevent CVD. We encourage the importance of nutrigenetics and nutrigenomics projects to explore the possible explanations of nutritional recommendations over TG reduction and cardiovascular risk from a molecular perspective in order to bridge the gap towards personalized care and accomplish better health improvements. Therefore, the scientific evidence on nutritional treatment to reduce TG should clarify exactly what to do with each population group, but also how to implement it in daily life.

## Figures and Tables

**Table 1:** Effect of diet on serum triglycerides.

Author, Year	Dietary Component	Dose/Time	Study Design n	Main Results on Triglyceride Levels
Kim, 2016 [30]	1. HMD 2. HWD	1. White bread, leg ham, and orange juice 2. Whole grain bread, chicken, milk, yogurt, and nuts−5, 0, 10, 20, 30, 40, 50, 60, 75, 90, 120, 150, and 180 min	Randomized crossover study, 64 overweight and obese adults (43 average age)	↓TG (iAUC)(−0.2, 0.5 mU/L/3 h for red meat/refined grain diet vs. 0.0, 0.4 mU/L/3 h for 12 of 17 dairy/chicken/nuts/wholegrain diet, *p* < 0.001)
Le, 2016 [29]	1. LFHC 2. LCHF 3. WRHFLC.	1. 20% fat, 65% carbohydrate, 15% protein 2. 35% fat, 45% carbohydrate, 20% protein 3. 35% fat, 45% carbohydrate, 20% protein 1 year	Unblinded clinical trial, 81 obese adults (mean age 43 years)	↓ TG in all study arms at 6 months (*p* < 0.05)
Johansen, 2017 [20]	1. Isocaloric diet 2. Regular diet	1. 55% carbohydrate (added sugars were less than 10%), 15% protein, and 30% fat 2. Consume their regular diet 2 days	Randomized trial, 11 overweight and obese men (aged 35–65 years)	↓ TG in diet group between the 2 study days 106.29 mg/dL (35.43–239.15) ^a^ 1.2 mmol/L (0.4–2.7) vs. 97.43 mg/dL (35.43–230) ^a^ 1.1 mmol/L (0.4–2.6)
Kim, 2017 [31]	1. HMD 2. HWD	1. Red meat, processed meat, refined grain diet, potato, indulgence food, vegetables, and fruits 2. Dairy, chicken, fish, legumes, nuts, whole grain, vegetables, and fruits 4 weeks per period	Randomized crossover study, 64 overweight and obese adults (43 average age)	↑ TG concentrations TG (*p* < 0.05) after HMD than after HWD
Maki, 2017 [32]	1. Combination of egg protein and UFA 2. Refined starches and added sugars	1. 42% carbohydrate, 23% protein and 35% fat 2. 58% carbohydrate, 15% protein and 27% fat >3 servings/d of study test foods (includes muffins, waffles, yogurt, cookies) 3 weeks intervention; 2 weeks washout period; 3 weeks intervention	Randomized, double-blind, controlled-feeding, crossover trial 25 subjects overweight or obese with HTG (21–70 years)	↓ TG in the egg protein and UFA intervention than during the refined-carbohydrate condition (−18.5% (−35.7, −6.9) ^c^ vs. −2.5 (−13.4,17.0) ^c^; *p* < 0.002)
Mateo-Gallego, 2017 [33]	1. Calorie-reduced diet 1 2. Calorie-reduced diet 2 3. Calorie-reduced diet 3	1. Protein 20%, carbohydrates 50%, fat 30% 2. Protein 27%, carbohydrates 43%, fat 30% 3. Protein 35% (80% from animal protein); carbohydrates 35%; fat 30%, 6 months	Randomized trial, 91 obese women (18–80 years)	↓ Levels TG in 35%-protein group at 3 months (*p* < 0.0001)
Saslow, 2017 [34]	1. VLCKD 2. PMD (ADA)	1. 20–50 g of non-fiber carbohydrates per day (eaten ad libitum) 2. A 9-inch plate: half the plate is filled with non-starchy vegetables, one-quarter with carbohydrates, and one-quarter with lean proteins, 32 weeks	RCT, 56 obese and type 2 diabetes adults (53 average age)	↓TG levels (EMM −60.1 mg/dL, 95% CI −91.3 to −28.9 mg/dL) in the intervention group, only at the 32-week time point (*p* = 0.01)
Veum, 2017 [28]	1. VHFLC 2. LFHC	1. 73% fat, 10% carbohydrate 2. 30% fat, 53% carbohydrate 12 weeks	Randomized trial, 56 overweight and obese men (aged 30–50 years)	Both groups improved dyslipidemia, with reduced circulating TG
Wright, 2017 [35]	1. LFPB 2. CD	1. 7% fat, 12% protein, and 81% carbohydrate (whole grains, legumes, vegetables, and fruits). Until satiation 2. Standard diet and general recommendations 12 months	RCT, 65 obese and type 2 diabetes adults (30–70 years)	Nonsignificant reduction in total TG
Bowen, 2018 [17]	1. DER 2. ADF + DER	1. 31% carbohydrate, 38% protein, 28% total fat (52% monounsaturated fat and 17% polyunsaturated fat). 2. Same macronutrient distribution as DER, fasting days −700 kJ/day, compared to the DER, and meal replacement Both groups were given meal replacements (approximately 1000 kJ, 25 g protein, 4 g fat, 27 g) 16 weeks	Randomized trial, 162 obese adults (25–60 years)	Both high-protein ADF + DER and DER programs were well accepted; TG improved (*p* < 0.05)
Moszak, 2018 [23]	Energy restricted diet	25–30% reduction in caloric dietary intake. Diet with an identical composition of macronutrients (especially proteins) derived from the same products. 20% protein, 25–30% from fat, and 50–55% from carbohydrates. 3 weeks	Unblinded clinical trial, 24 obese hospitalized adults (24–66 years)	↓ TG, −12.5% postintervention Not statistically significant
Øyri, 2018 [36]	1. Muffin 2. Muffin	1. 60 g/day (70% of fat, 40% SFA) 2. 60 g/day (70% of fat, 40% PUFA) 1 meal—3 to 5 weeks of washout	Randomized controlled double-blind crossover study 13 hypercholesterolaemic subjects with FH and 14 normolipidemic controls (18–30 years)	↔ Postprandial TG AUC with SFA meal 901.25 (612.5–1032.5) ^a^ mg/dL (10.3 (7.0–11.8) mmol/L compared with PUFA meal 586.25 (533.75–796.25)) ^a^ 6.7 (6.1–9.1) ^a^ in subjects with FH (*p* = 0.72) ↔ Postprandial TG iAUC with SFA meal 175 (78.75–288.75) mg/dL ^a^ (2.0 (0.9–3.3) mmol/L compared with PUFA meal 166.25 (87.5–253.75)) ^a^ 1.9 (1.0–2.9) ^a^ in subjects with FH (*p* = 0.36) ↔ Postprandial TG AUC and iAUC between groups (*p* = 0.56, *p* = 0.25)
Shah, 2018 [16]	1. HP 2. HMF	1. 840 kcal for men, 700 kcal for women (31.9% of protein, 15.5% of fat, 4.3% of MUFA, 9.9% of SFA, and 52.6% of carbohydrates) 2. 840 kcal for men, 700 kcal for women (35.2% of fat, 20.7% of MUFA, 12.6% of protein, 8.7% SFA, and 52.3% from carbohydrates) Postprandial (3 h) states	Randomized trial 24 overweight and obese adults (18–65 years)	↓ TG HP post-intervention at 120 min (geometric mean (95% CI): 148 (125–175) vs. 194 (164–230) mg/dL) and 180 min (167 (138–203) vs. 230 (189–278) mg/dL) HMF condition
Wade, 2018 [37]	1. MedDairy 2. LF	1. 3–4 servings daily of dairy foods, ≤1 serving of cheese, abundant use of extra virgin olive oil, ≥2–3 daily servings of fresh fruits, ≥3 weekly servings of legumes, ≥3 weekly servings of fish and seafood, ≥5 weekly servings of nuts or seeds, ad libitum consumption of whole-grain cereal 2. ≤20 mL oil/day and ≤2 teaspoons butter or margarine/day. Restricted foods: oil, butter, margarine, full-fat dairy products, processed and high-fat meats, nuts, chocolate, cakes, biscuits, pastry, and ice cream 8 weeks, and an 8-week washout period separating the interventions	Randomized, controlled, crossover trial, 41 overweight and obese adults (45–75 years)	↓ TG (mean difference: = −0.05 mmol/L; 95% CI: −0.08, −0.01 mmol/L; *p* < 0.01) after MedDairy intervention
Bowen, 2019 [38]	1. HOCO 2. Control oil 3. Canola oil	1. 19.1% MUFA, 7.0% PUFAs, 6.4% SFA 2. 10.5% MUFA, 10.0% PUFAs, 12.3% SFA 3. 17.5% MUFA, 9.2% PUFAs, 6.6% SFA 6 weeks (per period)	Double-blind, randomized, 3-period crossover, controlled feeding trial, 119 obese adults (aged 25–60 years)	↓TG levels (canola: 128.43 ± 3.54 mg/dL (1.45 ± 0.04 mmol/L) ^b^ *p* = 0.0182, HOCO: 127.55 ± 3.54 mg/dL (1.44 ± 0.04 mmol/L) ^b^ *p* = 0.0053, control: 124 ± 3.54 mg/dL (1.40 ± 0.04 mmol/L) ^b^ *p* = 0.0002)
Della Pepa, 2019 [39]	1. HPN2. LPN	1. Low in omega-3 (1.4 g/day) and rich in polyphenols (2.903 mg/day)Rich in omega-3 (4 g/day) and polyphenols (2.861 mg/day).2. Low in omega-3 (1.5 g/day) and polyphenols (365 mg/day)Rich in omega-3 (4 g/day) and low in polyphenols (363 mg/day)8 weeks	Randomized trial 88 overweight and obese men (35–70 years)	↓ TG decreased in HPM group (3.27 ± 2.20 vs. 2.55 ± 2.14 mmol/L × 6 h) ^b^
Gepner, 2019 [40]	1. LF 2. MED/LC	1. 30% of total fat, 10% SFA, 300 mg/day of cholesterol (whole grains, vegetables, fruits, and legumes and limited consumption of additional fats, sweets, and high-fat snacks) 2. Carbohydrate intake less than 40 g/day in the first 2 months, and thereafter a gradual increase up to 70 g/day (rich in vegetables and legumes and low in red meat, with poultry and fish and 28 g of walnuts/day) 18 months	Randomized controlled trial 278 dyslipidemia and obese adults (mean age 48 years)	The reduction in TG was similar between physical activity groups and MED/LC not statistically significant
Hutchinson, 2019 [22]	1. TRFe 2. TRFd	1. Eating window between 8 a.m. and 5 p.m. 2. Eating window between 12 p.m. and 9 p.m. Participants were asked to maintain their usual lifestyles, including sleep patterns, and to consume their habitual diet 1 week	Randomized, controlled, crossover trial, 50 overweight and T2DM risk adults (mean age 55 years)	↓ Fasting TG (*p* = 0.003) on day 7 versus day 0 in both TRF interventions
Kondo-Ando, 2019 [41]	1. LB 2. NB	1. 187.8 kcal: 20.7 g carbohydrate (dietary fiber 11.1 g); protein, 17.4 g; fat, 6.0 g 2. 185 kcal: 32.7 g carbohydrate (dietary fiber, 1.6 g); protein, 6.5 g; fat, 3.1 g. 7 days (1 and 2 h after the breakfast)	Unblinded clinical trial 41 T2DM hospitalized patients (20–80 years)	↓ TG 2 h after breakfast in the LB group compared with those in the NB group (*p* < 0.01)
Medina, 2019 [42]	1. DP 2. P	1. 500 kcal/day deficit 45–55% carbohydrate, 15–20% protein, 25–35% fat, < 7% SFA, 200 mg/day of cholesterol, 20–35 g fiber. Comprising high-fiber, polyphenol-rich and vegetable-protein functional foods: 14 g of dehydrated nopal, 4 g of chia seeds, 30 g of soy protein and 4 g of inulin) 2. Dehydrated sachets similar in kcal (28 g of calcium caseinate and 15 g of 137 maltodextrin) 3 months	Placebo-controlled, randomized, double-blind trial 81 overweight and T2DM adults (30–60 years)	↓ TG (*p* < 0.01) vs. the p group
Starr, 2019 [43]	1. HP 2. CG	1. 30% protein, 30% fat, 40% carbohydrate, with a protein intake of 1.2 g/kg 2. 15% protein, 30% fat, 55% carbohydrate diet with a protein intake meeting the RDA of 0.8 g/kg. 6 months	Randomized trial, 80 obese adults (64 average age)	↓ TG at endpoint; protein group had significant TG reduction (p ≤ 0.05) (−17.3 ± 50.2 mg/dL vs. 11.5 ± 34.7 mg/dL) ^b^
Shih, 2019 [44]	1. HLC 2. HLF	1. With respect to basal (−111.9 ± 76.1 carbohydrates, −506.7 ± 616.5 energy, 2.9 ± 4.4 change in saturated fatty acids) 2. With respect to basal (−36.1 ± 81.3 carbohydrates, −484.3 ± 625.8, −2.6 ± 3.6 change in saturated fatty acids) 12 months	Randomized trial, 609 obese adults (18–50 years)	↓TG in the context of a weight-loss study in which participants simultaneously decreased carbohydrate intake (*p* < 0.0001)
Tindall, 2019 [45]	1. WD 2. WFAMD 3. ORAD	1. 7% SFA, 16% PUFA, 3% ALA, 9% MUFA 2. 7% SFA, 16% PUFA, 3% ALA, 9% MUFA 3. 7% SFA, 14% PUFA, 0.5% ALA, 12% MUFA 6-week intervention, 22.8 ^c^ days washout period, 6-week intervention, 22.8 ^c^ days washout period, 6-week intervention	Randomized, controlled, 3-period, crossover, feeding trial, 36 subjects with overweight or obesity, LDL cholesterol between 128–177 mg/dL for men and 121–172 mg/dL for women and/or elevated brachial blood pressure (30–65 years)	↔ TG between-diet differences compared baseline 117.7 ± 8.1 mg/dL (1.34 ± 0.09 mmol/L) ^b^ to after intervention WD 116.5 ± 8.5 (1.33 ± 0.01) WFAMD 117.4 ± 8.1 (1.34 ± 0.09) ^b^ ORAD 118.1 ± 8.3 (1.35 ± 0.09) ^b^; *p* = 0.70
Haldar, 2020 [46]	1. Oil blend made with refined rice bran, flaxseed, and sesame oils 2. Oil blend made with refined rice bran, flaxseed, and sesame oils 3. Refined olive oil (control group)	1. 30 g/day (14.9% SFA, 52.2% PUFA, 32.9% MUFA) 2. 30 g/day (13.6% SFA, 58.4% PUFA, 28.1% MUFA) 3. 30 g/day (16.6% SFA, 12.5% PUFA, 70.8% MUFA) 8 weeks	Parallel-design, randomized, single-blind, controlled trial 131 borderline hypercholesterolemic (50–70 years)	↓ TG post-intervention time points (−10.3% ^d^; *p* < 0.0001) for all 3 intervention oils compared with baseline ↔ between the 3 treatments across time
Kim, 2020 [47]	1. BKD 2. TAD 3. 2010DGA	1. 64% carbohydrates, 15% protein, 21% fat 2. 48% carbohydrates, 15% protein, 37% fat 3. 53% carbohydrates, 16% protein, 31% fat 4 weeks per period	Three-period, randomized, crossover-controlled trial, in 62 overweight or obese adults	↓ TG significantly in three diets after 4 weeks (changes in TG levels) BKD: −33.11 ± 7.99 mg/dL ^b^ (0.37 ± 0.09 mmol/L) ^b^ (*p* < 0.001) TAD: −27.63 ± 7.99 md/dL (−0.31 ± 0.09 mmol/L) ^b^ (*p* = 0.001) 2010DGA: −20.69 ± 8.06 mg/dL (−0.23 ± 0.09 mmol/L) ^b^ (*p* = 0.012) No difference between treatment groups
Yokose, 2020 [48]	1. LFRC 2. MedRC 3. LCNRC	1. 1500 kcal per day for women and 1800 kcal per day for men (30% of fat, 10% of saturated fat, 300 mg of cholesterol) 2. 1500 kcal per day for women and 1800 kcal per day for men (no more than 35% of fat, 30 to 45 g of olive oil and nuts (5–7 nuts) per/day) 3. 20 g of carbohydrates per/day for the 2-month induction phase with a gradual increase to a maximum of 120 g per/day. The intakes of total calories, protein, and fat were not limited 24 months	RCT, 205 obese adults (40–65 years)	↓ TG in all three groups (*p* < 0.05 at 6 months) ↓ TG at both (MedRC and LCNRC) 6 and 24 months (all *p* for within-group comparison, 0.01)
Folwaczny, 2021 [49]	1. Isocaloric fat shake (SFA meal) 2. Isocaloric fat shake (MUFA meal) 3. Isocaloric fat shake (MCFA meal)	1. 80 g/day fat (68% SFA, 2% PUFA, 30% MUFA) 2. 80 g/day fat (8% SFA, 29% PUFA, 63% MUFA) 3. 80 g/day fat (99% SFA) 1 meal, 7 to 28 washout period, 1 meal, 7 to washout period	RCT 8 mildly hypertriglyceridemic (43 ± 12 y ^b^) and 5 normolipidemic controls (35 ± 5 y ^b^)	↓ iAUC TG with MCFA meal 152 ± 271 mg/dL*h ^b^ (1.73 ± 3.09 mmol/L*h) compared to SFA meal 1006 ± 583 (11.47 ± 665) ^b^; *p* = 0.03 and MUFA meal 962 ± 673 (10.97 ± 7.67) ^b^; *p* <0.01 in hypertriglyceridemic patients ↓ iAUC TG in normolipidemic controls with SFA meal 259 ± 160 mg*h/dL ^b^ (2.95 ± 1.82 mmol*h/L) and MUFA meal 248 ± 298 (2.83 ± 3.4) ^b^ compared to hypertriglyceridemic patients SFA meal 1006 ± 583 (11.47 ± 665) ^b^; *p* = 0.019, MUFA meal 962 ± 673 (10.97 ± 7.67) ^b^; *p* = 0.05.

HMD: high-meat diet; HWD: high-whole-grain diet; TG: triglycerides; AUC: area under curve; iAUC: incremental area under curve; LFHC: lower-fat higher-carbohydrate diet; LCHF: lower-carbohydrate higher-fat diet; LFPB: low-fat plant-based diet; CD: control diet; WRHFLC: walnut-rich, higher-fat, lower-carbohydrate diet; UFA: unsaturated fatty acids; HTG: hypertriglyceridemia; VLCKD: very low-carbohydrate ketogenic diet; PMD (ADA): plate method diet, American Diabetes Association; EMM: estimated marginal mean; CI: confidence interval; VHFLC: very high-fat low-carbohydrate; RCT: randomized clinical trial; DER: high-protein meal replacement program with daily energy restriction; ADF + DER: energy restriction adding alternate-day fasting and of modified-fasting and DER; FH: familial hypercholesterolemia; SFA: saturated fatty acids; HPN: high-polyphenol diet; HMF: high-monounsaturated fat; MUFA: monounsaturated fatty acids; PUFA: polyunsaturated fatty acids; MedDairy: MedDiet with 3–4 daily servings of dairy; LF: low fat; HOCO: high-oleic acid canola oil; HP: high-protein; LPN: low-polyphenol diet; MED/LC: combined Mediterranean and low-carbohydrate diets; TRF: time-restricted feeding; TRFe: time-restricted feeding early; TRFd: time-restricted feeding delay; T2DM: type 2 diabetes mellitus; LB: low-carbohydrate bread; NB: normal-carbohydrate bread; DP: dietary portfolio; P: placebo; CG: control group; RDA: recommended dietary allowance; HLC: healthy low-carbohydrate; HLF: healthy low-fat; WD: walnut diet; WFAMD: walnut fat acid-matched diet; ORAD: oleic acid–replaced-ALA diet; ALA: α-linolenic acid; LDL: low density lipoprotein; BKD: balanced Korean diet; TAD: typical American diet; 2010DGA: diet recommended by the 2010 Dietary Guidelines for Americans; LFRC: low-fat, restricted-calorie; MedRC: Mediterranean restricted-calorie; LCNRC: low-carbohydrate, non-restricted-calories; MCFA: medium-chain fatty acids; g/day: grams per day; mg: milligrams; mmol: millimoles; mU: milliunits, L: liter; dL: deciliter. ↓ Decrease; ↑ Increase; ↔ No significant difference ^a^ Values are reported on median (interquartile range) ^b^ Values are reported on mean ± SD ^c^ Values are reported on % of change medians (IQLs) ^d^ Values are % of change.

**Table 2:** Effect of nuts and flaxseed on serum triglycerides.

Author, Year	Dietary Component	Dose/Time	Study Design n	Main Results on Triglyceride Levels
Abbaspour, 2019 [58]	Mixed Nuts Pretzels	42.5 g/day 8 weeks	Randomized, parallel-arm controlled trial 54 overweight and obese subjects (18–55 years)	↓ TG levels post intervention from 0 to 8 weeks (101.5 ± 58.63 mg/dL (1.16 ± 0.67 mmol/L) ^a^ vs. 110.25 ± 96.25 (1.26 ± 1.1); *p* < 0.05). ↔ between groups (*p* = 0.655).
Gulati, 2017 [63]	Almond	Almonds substituted for 20% of total energy of fat 24 weeks	Pre-post intervention study design 50 T2DM patients	↓ TG post intervention with almond vs. baseline (170.5 mg/dL (72,499) (1.94 (0.68 mmol/L) ^c^ vs. 149.5 (72,499) (1.7 (0.83 mmol/L); *p* < 0.004).
Liu, 2017 [62]	Peanut shake Control	85 g per visit 10 days	Randomized, controlled, crossover trial 15 overweight or obese men (20–65 years)	IG vs. CG ↓ TG after 120 min (16,528.75 ± 1697.5 mg/dL (188.9 ± 19.4 mmol/L) ^a^ vs. 17,281.25 ± 1811.25 (197.5 ± 20.7)) and 240 min (16,616.25 ± 2126.25 mg/dL (189.9 ± 24.3 mmol/L)) vs. 17,263.75 ± 1610 (197.3 ± 18.4) with peanut shake and 240 min compared with control (*p* < 0.005).
Mah, 2017 [71]	Cashew (roasted, salted) Potato chips	28–64 g/day 32–64 g/day 4 weeks	Randomized, crossover, isocaloric, controlled-feeding study 51 hypercholesterolemic subjects (21–73 years)	↔ between IG and CG for TG from baseline (7.7% (−8.8, 17.3) ^b^ vs. 15.7 (1.1, 25.3); *p* = 0.206.
Mohan, 2018 [70]	Cashew nut	30 g/day 12 weeks	Parallel-arm, randomized controlled trial 269 T2DM subjects	↔ between groups in TG mean change post intervention (IG 4.3 ± 51.1 mg/dL (0.05 ± 0.58 mmol/L) ^a^ vs. CG 0.4 ± 62.2 (0 ± 0.71); *p* = 0.57).
Zibaeenezhad, 2017 [67]	PWO	15 mL 90 days	Randomized, double-blind, placebo-controlled clinical trial 100 hyperlipidemic and T2DM patients (35–75 years)	↓ TG (−15.04 mg/dL(−0.17 mmol/L); *p* = 0.021).
Akrami, 2018 [65]	FO SFSO	25 mL/day 7 weeks	Randomized controlled interventional trial 60 MS patients (30–60 years)	↓ TG levels after treatment with both oils IG: −52.46 ± 74.32 mg/dL (−0.6 ± 0.85 mmol/L) ^a^; *p* < 0.001 and CG: −53.46 ± 58.21 (0.61 ± 0.66 mmol/L; *p* < 0.001). ↔ compared between the two groups (*p* = 0.516).
Zibaeenezhad, 2019 [69]	Almond oil	10 mL, two times daily 30 days	Randomized open-label controlled clinical trial 99 hyperlipidemic adults	↔ post intervention with almond in TG (mean change): 6.01 ± 5.78 mg/dL (0.07 ± 0.07 mmol/L ^a^; 224.90 ± 57.93 (2.56 ± 0.66) vs. 131.05 ± 17.84 (1.49 ± 0.2); *p* = 0.491).
Coates, 2020 [61]	AED NFD with carbohydrate-rich snack foods	Portion of snack foods equivalent to 15% of their EER 12 weeks	Two-arm, parallel group randomized dietary intervention 128 overweight and obese adults (50–80 years)	↓ TG after AED compared to the CG (IG: 100.62 ± 5.25 mg/dL (1.15 ± 0.06 mmol/L) vs. 112.88 ± 6.13 (1.29 ± 0.07) ^a^; CG: 101.5 ± 0.61 (1.16 ± 0.007) vs. 03.25 ± 0.53 (1.18 ± 0.006); *p* = 0.008).
Costa e Silva, 2020 [68]	EVBNO SO	10 mL/day 10 mL/day 30 days	Randomized, double-blind, placebo-controlled clinical trial 31 MS patients (36–65 years)	↑ 68.6 ± 83.6 mg/dL (0.78 ± 0.95 mmol/L) ^a^ in TG (*p* < 0.05) after EVBNO consumption ↓ TG (−4.5 ± −4.5 mg/dL (0.05 ± 0.05 mmol/L); *p* = 0.8501) after SO Significant difference between EVBNO and SO (*p* < 0.05).
Yari, 2021 [66]	WFP HE FHE	30 g/day 1 g/day 30 g/ 1 g/day 12 weeks	RCT 173 adults with MS	↓ TG after 12 weeks with WFP and FHE group intervention (211.0 ± 83.9 mg/dL (2.41 ± 0.96 mmol/L) vs. 145.0 ± 58.5 (1.65 ± 0.67) ^a^; *p* < 0.001). ↓ TG in WFP compared to control (*p* < 0.002).
Ghanavati, 2021 [59]	NELCD NFLCD	8 weeks	RCT 67 overweight and obese subjects (aged 58.8 ± 7.4 years)	↓ TG in both groups compared with baseline (mean change) (IG: −39.7 mg/dL (−0.45 mmol/L) vs. CG −33.6 (0.38); *p* = 0.60). ↔ between groups.
Haldar, 2020 [46]	RFSSO	30 g per day 8 weeks	Parallel-design randomized controlled trial 143 hypercholesterolemic subjects	↓ TG after intervention (−10.3%; *p* < 0.001).
Julibert, 2020 [60]	MD enriched with nuts	MD + 30 g/d of nuts (almond, pistachios, walnuts) MD + 1 L/week EVOO 1 year	Randomized trial 5800 overweight, obese and MS adults (55–75 years)	↓TG (−7.8 ± 68.9 mg/dL (0.09 ± 0.79 mmol/L) ^a^ after 1 year of nut consumption Greater TG ↓ in T3 compared with T1 (−10.6 ± 60.1 mg/dL (0.12 ± 0.69 mmol/L) vs. −4.1 ± 77.0 (0.05 ± 0.88); *p* < 0.005).
Kuang, 2020 [64]	DFF Biscuit	100 g/day 60 days	Double-blind randomized controlled trial 53 overweight and obese patients (16–36 years)	Difference between groups after 60 days of intervention (*p* < 0.005). ↓ TG compared day 0 to day 60 DFF group (105 ± 51.63 mg/dL (1.20 ± 0.59 mmol/L) ^a^ vs. 101.5 ± 55.15 (1.16 ± 0.63); *p* < 0.005)

TG: triglycerides; T2DM: Type 2 diabetes mellitus; IG: intervention group; CG: control group; PWO: persian walnut oil; FO: flaxseed oil; SFSO: sunflower seed oil; MS: metabolic Syndrome; EVBNO: extra virgin brazil nut oil; SO: soybean; WFP: whole flaxseed powder; HE: hesperidin; FHE: flaxseed and hesperidin; AED: almond-enriched diet; NFD: nut free diet with oil carbohydrate-rich snack foods; EER: estimated energy requirements, NELCD: nuts-enriched, low-calorie diet; NFLCD: nuts-free, low-calorie diet; RCT: randomized clinical trial; RFSSO, refined rice bran, flaxseed, and sesame oils; MD: Mediterranean diet; EVOO: extra virgin olive oil; DFF, defatted flaxseed flour (biscuits with flaxseed meal; g/day: grams per day; mg: milligrams; mmol: millimoles; L: liter; dL: deciliter. ↓ Decrease; ↑ Increase; ↔ No significant difference ^a^ Values are reported on mean ± SD ^b^ Values are medians (95% CIs) ^c^ Values are median and range.

**Table 3:** Effect of soy on serum triglycerides.

Author, Year	Dietary Component	Dose/Time	Study Design n	Main Results on Triglyceride Levels
Dong, 2016 [75]	PSSMP NPSSMP	30 g/day (2 g plant sterol) 30 g/day 6 months	RCT 137 subjects with mild to moderate hypercholesterolemia (55–65 years)	↔ between the NPSSMP and PSSMP group in TG after 3 and 6 months. (Baseline, IG: 166.25 ± 85.75 mg/dL (1.90 ± 0.98 mmol/L) and CG: 171.5 ± 84 (1.96 ± 0.96); *p* = 0.704). After 6-month IG: 147 ± 89.25 mg/dL (1.68 ± 1.02 mmol/L) vs. CG: 163.63 ± 64.75 (1.87 ± 0.74); (*p* = 0.236).
Lu, 2019 [77]	Shake mix MRDSP-SF-CR	2 times/day 29 g: 6.3 g from protein (21% calories) 15 g from carbohydrates (of which 5 g are dietary fiber, 50% calories) 3.9 g from fats (29% calorie) 8 weeks	RCT 50 obese subjects (20–80 years)	↓ 11.5% after intervention compared with baseline (*p* < 0.05).
Tabrizi, 2019 [78]	SBL PC *	4 capsules 335/50 mg/day 4 capsules (1240/72 mg) 8 weeks	Triple-blind, RCT crossover 31 participants with cardiovascular risk factors (dyslipidemia or obesity or hypertension or HTG) (33–75 years)	↔ in TG after 8 weeks of SBL treatment. ↓ TG without significance after intervention in PC (8.0 (−17.00, 1.00) ^a^; *p* = 0.089).
Oliveira, 2020 [76]	SMPS SM	400 mL/day + 1.6 g 400 mL/day 4 weeks	Controlled, crossover study 38 moderately hypercholesterolemic subjects (38–77 years)	↓ 8.3% on TG levels after SMPS (*p* < 0.05) Difference after 4 weeks with SMPS compared SM (133 ± 7 mg/dL (1.52 ± 0.08 mmol/L) vs. 154 ± 10 (1.76 ± 0.11); *p* = 0.008).

PSSMP: phytosterol ester soy milk powder; NPSSMP: not phytosterol ester soy milk powder; RCT: randomized clinical trial; TG: triglycerides; CG: control group; MRDSP-SF-CR: meal replacement diet with rich soy/pea protein, soluble fibers and calorie restriction; SBL, soybean lunasin; PC, placebo capsule; HTG: hypertriglyceridemia; SMPS: soy milk with phytosterol; SM: soy milk; g/day: grams per day; mg: milligrams; mmol: millimoles; L: liter; dL: deciliter. ↓ Decrease; ↑ Increase; ↔ No significant difference. Values are reported on mean and standard deviation; ^a^ Values are difference and 95% CIs; * Corn Bran 230, fiber plus, LLC and protein.

**Table 4:** Effect of other foods on serum triglycerides.

Author, Year	Dietary Component	Dose/Time	Study Design n	Main results on Triglyceride Levels
Fernández-Castillejo, 2016 [80]	1. VOO 2. FVOO 3. FVOOT	1. (25 mL/day) 2. (25 mL/day 3. (25 mL/day) 3 weeks and 2 week washout period	Randomized, double-blind, crossover, controlled trial 33 hypercholesterolemic subjects	↔ between groups in TG after 3 weeks and 2-week washout period (1) VOO −7.9 (−25 to 9.1 mg/dL) ^a^, (2) FVOO 3.8 (−14 to 21), (3) FVOOT 3.8 (−14 to 22).
Lee, 2016 [81]	1. LFM 2. CG	1. Two packs of low-fat milk per day, 200 mL twice daily 2. Maintain habitual diet 6 weeks	Parallel-group dietary intervention study, 58 overweight and metabolic syndrome subjects	↔ between groups in TG after 6 weeks IG: 35.9 ± 101.5 mg/dL ^b^ vs. CG 17.2 ± 156.8; *p* = 0.301.
Suganya, 2017 [83]	1. Standard statin therapy 2. Nutritional supplement in addition to the standard statin therapy.	Nutritional supplement (mix spices: cuminum, piper nigrum, solanum nigrum) 9 months	Clinical trial two arms, 65 dyslipidemic subjects	↓ TG levels after supplement intake along with standard therapy, showed significant decrease in TG (338.2 ± 256.1 mg/dL ^b^ to 216.9 ± 102.6 mg/dL); *p* < 0.005.
Sarriá, 2018 [86]	1. IG 2. CG	1. 3 servings/day of the blend (510.6 mg hydroxycinnamic acids and 121.2 mg caffeine/day) 2. Control drink 8 weeks	Crossover, randomized, controlled study, 52 hypercholesterolemic and normocholesterolaemic	↓ TG levels (*p* = 0.017) and the reduction were much greater in the hypercholesterolemic subjects (group effect, *p* = 0.027).
Franck M, 2020 [85]	1. IG 2. CG	1. 280 g/day of frozen raspberries 2. Maintain usual diet 8 weeks	RCT, trial, 54 overweight/ obese subjects	No significant differences between follow-up and baseline values between CG and IG for triglycerides (Baseline: CG 1.42 ± 0.61 ^b^, IG 1.47 ± 0.82 vs. wk8 CG 1.38 ± 0.61, IG 1.29 ± 0.61; *p* = 0.71).
Koutsos, 2020 [84]	1. WA 2. CB	1. 2 apples/day 2. Sugar and energy-matched apple control beverage 8 weeks (4-week washout period)	Randomized, controlled, crossover, intervention study, 40 hypercholesterolemic subjects	↓ TG levels WA (WA: 1.17 mmol/L; CB: 1.30 mmol/L; *p* = 0.021).
Petersen, 2020 [82]	HSFHC	1. 0 g spices 2. 2 g of spices 3. 6 g of spices	randomized, controlled, crossover, pilot study, 13 overweight/ obese men	↓ TG 2 g of spices vs. the no spice meal (−18 ± 6 mg/dL ^b^; *p* = 0.015); no difference was observed between the meal with 6 g of spice and the no
Santos, 2020 [79]	1. EVOO group 2. TBD 3. TBD + EVOO	1. 52 mL/day of EVOO 2. Common Brazilian foods 3. 52 mL/day of EVOO 12 weeks	RCT 149 subjects with several obesity	↔ between groups in TG after 12 weeks (1) EVOO group (160.46 ± 79.85 mg/dL ^b^ vs. 151.35 ± 65.53; *p* = 0.296), (2) TBD (154.75 ± 87.76 vs. 142.46 ± 64.96; *p* = 0.069), (3) TBD + EVOO (165.60 ± 67.72 vs. 160.83 ± 77.33; *p* = 0.286).

VOO: virgin olive oil; FVOO: functional virgin olive oil; FVOOT: functional virgin olive oil with thyme; TG: triglycerides; LFM: low-fat milk; CG: control group; IG: intervention group; LFM; low-fat muffins; RCT: randomized clinical trial; WA: whole apple; CB: apple control beverage; HSFHC: high-saturated-fat, high-carbohydrate meal; EVOO, extra virgin olive oil; TBD: traditional Brazilian diet; g/day: grams per day; mg: milligrams; mmol: millimoles; L: liter; dL: deciliter. ↓ Decrease; ↑ Increase; ↔ No significant difference. ^a^ Values are change and (95% CIs). ^b^ Values are reported on mean and standard deviation.

**Table 6:** Effect of flavonoids on serum triglycerides.

Author, Year	Dietary Component	Dose/Time	Study Design n	Main results on Triglyceride Levels
Gutiérrez-Salméan, 2016 [110]	EPIC Placebo	Two capsules of 25 mg EPIC or placebo 4 weeks	Randomized, placebo-controlled, double-blind study 30 hypertriglyceridemia subjects (18–55 years)	↓ TG by 75 mg/dL (0.86 mmol/L) (26.5%, *p* < 0.05), while in the placebo group it decreased by 40 mg/dL (0.46 mmol/L) (14.7% *ns*). ↓ TG after EPIC group compared baseline values (207 ± 73.2 mg/dL (2.36 ± 0.83 mmol/L) vs. 282 ± 94.4 (3.21 ± 1.08); *p* = 0.008).
Ohara, 2016 [112]	GH GHC Placebo	3 tablets 500 mg/day 25, 50, 75 mg/day 12 weeks	Randomized double-blind placebo-controlled trial 75 overweight and obese subjects (25–65 years)	↓ TG after 8 weeks of intervention with GH + 75 mg of caffeine 29.3 ± 66 mg/dL (0.33 ± 0.75 mmol/L). ↔ TG after 4-, 8- and 12-weeks post intervention in all groups.
Tariq, 2016 [123]	CZRHT	Twice daily G1: 500 mg G2: 1 g G3: 1.5 g in 200 mL water 2 months	Randomized trial 30 hypercholesterolemic male (25–40 years old)	↓ TG of 12.5% after 60 days for G3 compared with day 0 (147.61 ± 2.56 mg/dL (1.68 ± 0.03 mmol/L) vs. 168.70 ± 2.92 (1.92 ± 0.03); *p* < 0.05).
Dicks, 2018 [124]	CP CFC	Capsules of 2.5 g/day of flavanol-rich cocoa (207.5 mg) or CFC 12 weeks	Double-blind randomized, placebo-controlled trial 42 hypertensive and T2DM patients (median of 64.2 years)	↔ TG after CP in either group compared with baseline (IG: 122.5 (78.75, 157.5 mg/dL) 1.4 (0.9, 1.8 mmol/L) ^b^ vs. 113.75 (78.75, 166.25) 1.3 (0.9;1.9) and CFC: 131.25 (96.25, 175 mg/dL) 1.5 (1.1, 2.0 mmol/L) vs. 157.5 (113.75, 201.25) 1.8 (1.3; 2.3); *ns*).
Kirch, 2018 [111]	EPIC Placebo	25 mg (encapsulated) 2 weeks intervention 2 weeks washout	Randomized, placebo-controlled, double-blind crossover study 48 overweight or obese and MS adults (20–65 years)	↔ TG after intervention with EPIC compared with week 0 (195.13 ± 23.63 mg/dL (2.23 ± 0.27 mmol/L) ^a^ vs. 194.25 ± 16.62 (2.22 ± 0.19); *ns*) ↔ after intervention and time.
Leyva-Soto, 2018 [109]	DC MC	2 g/day (70% cocoa or 2 g of MC) 6 months	Randomized, placebo-controlled, double-blind study 84 obese, dyslipidemia and T2DM young subjects (men and women, 23.8 and 23.6, respectively).	↓ TG after 6 months with DC intervention compared with baseline (153.26 ± 18.95 mg/dL (1.75 ± 0.22 mmol/L) ^a^ vs. 228.25 ± 17.9 (2.6 ± 0.2); *p* < 0.05).
Braxas, 2019 [9]	GE Placebo	2 capsules daily (54 mg of GE or placebo capsule) 12 weeks	Randomized, double-blind, placebo-controlled clinical trial 54 post-menopausal women with T2DM (47–69 years)	↓ TG in genistein group compared with baseline values (−10.19%; *p* < 0.05). ↓ TG after intervention with genistein compared with placebo (IG: 162.14 ± 52.04 mg/dL (2.11 ± 0.59 mmol/L) vs. 197.42 ± 76.44 (2.25 ± 0.87) and CG: 194.46 ± 77.80 mg/dL (2.22 ± 0.89) vs. 195.69 ± 63.41 (2.23 ± 0.72); *p* < 0.05).
Rynarzewski, 2019 [125]	CP Placebo	2.5 g of flavonoid-rich cocoa or microcrystalline cellulose Unique dose (postprandial) 2 weeks washout	Randomized, placebo-controlled, double-blind crossover study 12 T2DM, obese and hypertensive adults (>18 years)	↔ TG postprandial after CP treatment compared with baseline (4 h: 168.88 ± 16.63 mg/dL (1.93 ± 0.19 mmol/L) ^a^ vs. 0 h: 168.88 ± 19.25 (1.93 ± 0.22); *p* = 0.184).
Yari, 2020 [126]	HE Placebo	2 capsules daily of 500 mg or placebo 12 weeks	Randomized, double-blind, placebo-controlled clinical trial 49 MS patients (18–70 years)	↓ TG after HE intervention compared with placebo (IG: 185.50 ± 84.34 mg/dL vs. 136.41 ± 72.86) (CG: 155.61 ± 54.12 vs. 164.44 ± 63.77; *p* < 0.05).
Yousefi, 2021 [113]	GSE + RCD Placebo	3 capsules of 300 mg/day or placebo capsules 250 kcal lower than EER 12 weeks	Randomized, placebo-controlled trial 40 obese or overweight individuals (20–50 years)	↓ TG after 12 weeks in GSE group compared with baseline (−33.73 ± 14.07 mg/dL (−0.38 ± 0.16 mmol/L); *p* < 0.05).

EPIC: epicatechin cocoa; TG: triglycerides; GH: glucosyl hesperidin; GHC: glucosyl hesperidin with caffeine; CZRHT: Curcuma zedoaria roscoe herbal tea; G1–3: group 1–3; CP: cocoa powder; CFC: cocoa free capsules; T2DM: type 2 diabetes mellitus; IG: intervention group; CG: control group; DC: dark chocolate; MC: milk chocolate; GE: genistein; HE: hesperidin; GSE + RCD: grape seed extract and restricted calorie diet; EER, estimated energy requirement; MS: metabolic syndrome; *ns*: non-significant; g/day: grams per day; mg: milligrams; mmol: millimoles; L: liter; dL: deciliter; ↓ decrease; ↑ increase; ↔ No significant difference. Values are represented as means (SD). ^a^ Values were presented as means (SEM). ^b^ Values were presented as median (25th–75th centile).

**Table 7:** Effect of dietary fiber on serum triglycerides.

Author, year	Dietary Component	Dose/Time	Study Design n	Main Results on Triglyceride Levels
Dodevska, 2016 [134]	LFHF RSD	25–35 g/day of fiber 25–35 g/day of fiber Plant foods replaced with RSD 12 months	Experimental Trial 47 overweight, obese and dyslipidemia adults	↑ TG (2.8%) after 12 months of LFHF group. ↔ in TG on LFHF group compared with RSD (*p* = 0.717).
Li, 2016 [131]	WGO LFHFHD 7-day cyclical menu	50 g 100 g 30 days	RCT 298 overweight and T2DM participants	↓ TG after 50 g WGO compared with baseline, 180.25 mg/dL (92.75) (2.06 mmol/L (1.06)) ^a^ vs. 173.25 mg/dL (144.38) (1.98 mmol/L (1.65)). Change −7.88 mg/dL (−27.13, −11.38) (−0.09 mmol/L (−0.31, −0.13); *p* = 0.003) ^a,b.^ ↓ TG after 100 g WGO compared with baseline, 136.5 mg/dL (67.38) ^a^ (1.56 mmol/L (0.77)) ^a^ vs. 173.25 mg/dL (87.5) ^a^ (1.98 mmol/L (1.00) ^a^). Change −37.63 mg/dL (−56.88, −18.38) ^a^ (−0.43 mmol/L (−0.65, −0.21) ^a^; *p* = 0.003). *p* value between groups (change).
Raimondi de Souza, 2016 [138]	Oat bran Corn starch and rice flour 40 g (placebo group)	40 g/day (3 g of beta-glucans) 40 g/day 90 days	Double-blind, placebo-controlled, block-randomized trial 132 hypercholesterolemic patients (>20 years)	↓ TG in the oat bran group compared baseline to 30 days after intervention, 147 mg/dL (106–232) ^a^ 1.68 mmol/L (1.21–2.64) vs. 121 (93–178) (1.38 (1.06–2.03) ^a^; *p* < 0.005) ↓ TG in the oat bran group compared with the placebo group; *p* < 0.005
Tessari, 2016 [133]	FB RWB	Fiber (7 g/100 g) Beta glucan/starch ratio of (7.6:100, g/g) 6 months	Observational, controlled study with parallel groups T2DM (50–80 years).	↓ TG at end of FB compared with RWB (140 ± 14 mg/dL (1.60 ± 0.16 mmol/L) ^d^ vs. 95.38 ± 10.5 (1.09 ± 0.12); *p* = 0.16).
Vetrani, 2016 [132]	Isoenergetic diet: WGCF ^c^ RCP	52% carbohydrates 46% GI 72% GI 12 weeks	Randomized, controlled, parallel-group design 54 overweight, obese, and MS adults (men and women, median 58.4 and 57.2, respectively).	↓ TG after 12 weeks of intervention on WGCF (−16.9 ± 2.8 mg/dL (0.19 ± 0.03 mmol/L); *p* = 0.538) ^d^. ↓ TG at the end in the WGCF compared to the control (*p* < 0.005).
Gulati, 2017 [139]	Oats in the form of porridge and upma Routine diet and usual exercise habits (control group)	70 g/day 4 weeks	Prospective, randomized, parallel, controlled study 69 subjects mildly hypercholesterolemic (20–50 years)	↔ TG post intervention in both groups Oats group: 162.2 ± 54.7 mg/dL ^b^ (1.85 ± 0.62 mmol/L) to 172.7 ± 75.2 (1.97 ± 0.86) Control group: 163.6 ± 62.5 mg/dL (1.87 ± 0.71 mmol/L) to 165.8 ± 69.2 (1.89 ± 0.79) ↔ % reduction TG post intervention between groups 5.9 (−29.6, 41.5) ^a^; *p* = 0.03.
Pal, 2017 [108]	Fiber Supplements: PSY PGY RF	5 g/day each one 12 months	Randomized, double blind, parallel study 159 overweight and obese people (19–68 years)	↓ TG in the PSY after 6 months (100.62 ± 0.53 mg/dL (1.15 ± 0.006 mmol/L) ^e^ vs. 108.5 ± 0.53 (1.24 ± 0.006); *p* < 0.05) compared to baseline.
Anggadiredja, 2018 [136]	FMC IF	Twice daily 4 g of soluble fiber 0.5 g of insoluble fiber 6 weeks	Double-blind, randomized parallel-group study 41 hypercholesterolemic participants (>18 years).	↔ Between FMC and IF groups at start or at the end of the study (104.90 ± 9.9 mg/dL (1.24 ± 0.11 mmol/L) ^d^ vs. 113.95 ± 12.65 mg/dL (1.3 ± 0.14); *p* > 0.5 and 96.2 ± 9.99 mg/dL (1.1 ± 0.11 mmol/L) ^d^ vs. 102.4 ± 11.95 (1.17 ± 0.14) ^d^; *p* > 0.05).
Babiker, 2018 [135]	GA Placebo (pectin)	30 g 5 g 3 months	Randomized, double-blind, placebo-controlled clinical trial 91 T2DM subjects (mean 50.09 years)	↔ TG post GA intervention vs. baseline (*p* = 0.17) ↔ between GA and control group after intervention (*p* = 0.958).
Schioldan, 2018 [141]	HCD WSD	64 g/day dietary fiber 18 g/day dietary fiber 4 weeks intervention, 4 to 6 weeks washout period, 4 weeks intervention	Open-label, randomized, crossover study 19 subjects with MS (39–75 years)	↔ TG compared baseline to post intervention in the diets: WSD: 118.13 (90.13–157.5 mg/dL) ^e^ (1.35 (1.03–1.8 mmol/L)) vs. 138.25 (97.13–175) (1.58 (1.11–2.0)); *p* = 0.58 HCD: 122.5 (90.13–164.5 mg/dL) ^e^ (1.4 (1.03–1.88 mmol/L)) vs. 136.5 (88.38–173.25) (1.56 (1.01–1.98)); *p* = 0.67.
Fatmah, 2020 [114]	HFB *TB*	100 g/day 100 g/day 4 weeks	Quasi-experimental design, single-blind, randomized controlled trial 66 T2DM participants (50–60 years)	↓ TG after caromma biscuit intervention (123.67 ± 73.69 mg/dL (1.41 ± 0.84) ^e^ vs. 175.09 ± 112.64 (2 ± 1.28) ^e^; *p* <0.001).
Rakvaag, 2019 [115]	WPLF MDLF WPHF MDHF	2 daily servings with 300 mL of water + 10 g/day from fiber 2 daily servings with 300 mL of water + 30 g/day from fiber 12 weeks	Double-blind, randomized parallel-group study 41 hypercholesterolemic adults (men and women, median 42 and 38.6, respectively).	Fasting TG ↓ TG after intervention with WPLF compared with baseline (10,237.5 ± 2887.5 mg/dL (117 ± 33 mmol/L) ^e^ vs. 10,237.5 ± 1925 (117 ± 22); *p* < 0.05). Postprandial TG WPLF ↓ TG tAUC (360 min) after 12 weeks compared with baseline (19,512.5 ± 8662.5 mg/dL (223 ± 99 mmol/L) ^e^ vs. 24,150 ± 11,550 (276 ± 132); *p* <0.05). WPHF ↓ TG tAUC after intervention vs. baseline (18,200 ± 5687.5 mg/dL (208 ± 65 mmol/L) ^e^ vs. 20,300 ± 8312.5 (232 ± 95); *p* < 0.05). ↔ between WPHF and the two MD groups.
Cicero, 2020 [140]	Proprietary formulation of beta-glucans Oat-based isocaloric placebo without beca-glucan	3 g/day beta-glucans of oat 2 months intervention-4 weeks wash-out period- 2 months intervention	Double-blind, placebo-controlled, crossover randomized clinical trial 83 free-living subjects, adherent to Mediterranean diet, with moderate hypercholesterolemia (20–65 years)	↔ TG compared baseline to post intervention in oat with beta-glucan intervention 129.5 ± 70.88 mg/dL ^e,b^ (1.48 ± 0.81 mmol/L) vs. 141.75 ± 96.25 ^e^ (1.62 ± 1.10); *p* = 0.385.

LFHF: low-fat, high-fiber diet; TG: triglycerides; RSD: resistant starch rich diet*; WGO: whole grain oat; LFHFHD: low-fat and high-fiber healthy diet; RCT: randomized clinical trial; T2DM: type 2 diabetes mellitus; FB: functional bread; RWB: regular white bread; WGCF: whole grain cereal foods; MS: metabolic syndrome; RCP: refined cereal products; PSY: psyllium; PGY: polyglycoplex; RF: rice flour; FMC: fiber multivitamin combination; IF: insoluble fiber; GA: gum arabic; HCD: healthy carbohydrate diet; WSD: refined-carbohydrate western-style diet; HFB: high-fiber biscuits; TB: *temma biscuit*; WPLF: whey protein low fiber; MDLF: maltodextrin low fiber; WPHF: whey protein high fiber; MDHF: maltodextrin high fiber; tAUC: total area under the curve; g/day: grams per day; mg: milligrams; mmol: millimoles; L: liter; dL: deciliter. *Resistant starch includes: cooked rice, cooked potatoes, cooked beans, cooked peas, cooked lentils, cooked millet, pasta, polenta, rye and barley flakes, pumpkin, pearl barley, sweet corn, sourdough bread, rye bread, and green bananas. ↓ Decrease; ↑ Increase; ↔ No significant difference. ^a^ Values were presented as means (95% CI). ^b^ Adjusted for sex, age, drinking, smoking, PAL, education, family history of DM, and medication in the analysis of covariance model. ^c^ Whole grain diet including whole wheat bread, whole wheat pasta, barley kernels, whole grain oat biscuits and breakfast cereal (all bran and flakes). Control diet contained commercial products based on refined cereal (wheat bread, rice, pizza, cornmeal porridge, and breakfast cereal (rice krispies). ^d^ Values were presented as means (SEM). ^e^ Values were presented as means (SD).

**Table 8:** Effect of genetic variants or gene expression on serum triglycerides.

Author, Year	Dietary Component	Dose/Time	Study Design n	Main Results on Triglyceride Levels
Corella, 2016 [152]	Mediterranean diet + Nuts Mediterranean diet + EVOO LF diet	30 g/day 1 L per week 4.8 years ^a^	Large, multicenter, randomized and controlled clinical trial 7098 with T2DM or cardiovascular risk genotyped for *CLOCK* rs4580704 C>G (55–80 years)	↔ TG in any genotype or diet.
De Luis, 2016 [142]	HP diet S diet	1050 kcal/day, 33% fats, 33% carbohydrates, and 34% proteins 1093 kcal/day, 27% fats, 53% carbohydrates, and 20% proteins 9 months	RCT 283 obese subjects genotyped for *UCP3* rs1800849 C>T (52.9 ± 11.2 y ^a^ and 52.3 ± 10.4)	↓ TG with HP diet and only in C/C genotype, 122.1 ± 31.1 mg/dL ^a^ (1.39 ± 0.35 mmol/L) to 106.6 ± 13.2 (1.22 ± 0.15) at 9 months to baseline—15.5 ± 3.9 (0.15 ± 0.04) ^a^; *p* < 0.05.
De Luis, 2017 [143]	Low in carbohydrates and hypocaloric diet LF and hypocaloric diet	1507 kcal/day 36% fats, 38% carbohydrates, 26% proteins 1500 kcal/day, 27% fats, 53% carbohydrates, 20% proteins 3 months	RCT 283 obese subjects genotyped for *NPY* rs16147 G>A (46.8 ± 10.1 y ^a^).	↔ TG in any genotype or diet.
De Luis, 2017 [149]	High-PUFA hypocaloric diet High-MUFA hypocaloric diet	1448 kcal/day 45.9% carbohydrates, 34.3% lipids (21.8% SFA, 55.3% MUFA, 22.9% PUFA), and 19.8% proteins 1442 kcal/day 46.0% carbohydrates, 34.4% lipids (21.6% SFA, 67.7% MUFA, 10.7% PUFA), and 19.6% proteins.	RCT 361 Obese subjects *BDNF* rs10767664 A>T (20–65 years).	↔ TG in any genotype or diet.
Arpón, 2018 [153]	Mediterranean diet + Nuts Mediterranean diet + EVOO LF diet	30 g/day 1 L per week 5 years	RCT 36 participants with MS (60 and 70 years)	↔ TG with any diet.
De Luis, 2018 [150]	Standard hypocaloric diet	1520 kcal/day 52% of carbohydrates, 25% of lipids and 23% of proteins (50.7% MUFA, 38.5% SFA and 11.8% PUFA) 3 months	Non-randomized interventional study 80 obese subjects with the genetic variant *BDNF* rs10767664 A>T (20–65 years)	↓ TG in non T allele carriers −13.2 ± 7.5 mg/dL ^a^ (−0.15 ± 0.09 mmol/L) compared to T allele group +2.8 ± 1.2 ^a^ (0.03 ± 0.01) *p* = 0.02.
Griffin, 2018 [145]	High-MUFA and low-glycaemic diet High-MUFA and high-glycaemic diet LF and low-glycaemic diet LF and high-glycaemic diet High-SFA high-glycaemic diet (reference diet)	38% fats (10% SFA), 45% carbohydrates 38% fats (10% SFA), 45% carbohydrates 28% fats (10% SFA), 55% carbohydrates 28% fats (10% SFA), 55% carbohydrates 38% fats (16% SFA), 45% carbohydrates All diets are iso-energetic 24 weeks	Secondary analysis of data from a five-arm, randomized controlled, parallel dietary intervention trial 389 subjects with increased risk of developing the metabolic syndrome and genotyped for *APOE* rs429358 (112) and rs7412 (158) (E2/E2, E2/E3, E4/E4, E3/E4 and E3/E3 genotypes) (30–70 years)	↑ TG Among carriers of E2 40.25 mg/dL ^b^ (0.46 mmol/L) *p* = 0.001 and E4 28.88 (0.33); *p* = 0.01 compared with E3/E3 when SFA was replaced with MUFA and low glycemic index carbohydrates.
Aller, 2019 [147]	High-MUFA and hypocaloric diet High-PUFA and hypocaloric diet	34.1% fats (21.7% SFA, 67.5% MUFA, 22.7% PUFA), 46.6% carbohydrates and 19.2% proteins 34.4% fats (21.8% SFA, 55.5% MUFA, 10.8% PUFA), 45.7% carbohydrates and 19.9% proteins 3 months	RCT 362 obese subjects genotyped for *CB2R* rs3123554 G>A (25–65 years)	↓ TG after high MUFA diet in G/G genotype 129.8 ± 9.1 mg/dL ^a^ (1.48 ± 0.1 mmol/L) to 110.7 ± 10.0 (1.26 0.11); *p* = 0.03.
De Luis, 2020 [146]	High-MUFA and hypocaloric diet High-PUFA and hypocaloric diet	34.1 fats, 46.6% carbohydrates and 19.2% proteins 34.4% fats, 45.7% carbohydrates and 19.9% proteins 400–500 kcal/day less than the individually estimated total energy expenditure in both diets 3 months	RCT 361 obese subjects genotyped for *MTNR1B* rs10830963 C>G (18–70 years)	↔ TG in any genotype or diet.
Franck, 2020 [85]	Frozen raspberries Maintain usual diet (control group)	240 g/day 8 weeks	RCT 54 subjects with overweight or abdominal obesity (18–45 years)	↔ TG between groups.
Merino, 2020 [151]	Intensive lifestyle intervention Metformin Placebo	Healthy low-calorie, low-fat diet Started at a dose of 850 mg/day and increased to 1700 mg/day 1 year	Multicenter randomized controlled trial 2658 participants with glucose intolerant and polygenic risk score of 204 variants representative of 160 coronary artery disease loci (50.7 years ^a^)	↓ TG with the intensive lifestyle intervention −14.79 (−16.89, −12.69) ^c^ mg/dL (−0.169 (−0.193, −0.145)) mmol/L compared to placebo −5.25 (−7.35, −3.15) (−0.060 (−0.084, −0.036)) *p* < 0.001.
Primo, 2020 [148]	HP diet S diet	1050 kcal/d, 33% fats, 33% carbohydrates, and 34% protein 1093 kcal/d, 27% fats, 53% carbohydrates, and 20% protein 9 months	RCT 238 obese subjects genotyped for *CB2R* rs3123554 G>A (53.9 ± 9.1 y ^a^)	↓ TG in G/G genotype with the HP diet 125.8 ± 23.1 mg/dL ^a^ (1.43 ± 0.26 mmol/L) to 104.1 ± 13.2 (1.19 ± 0.15) and with S diet 129.1 ± 22.6 (1.47 ± 0.26) to 114.1 ± 20.1 (1.3 ± 0.23) at 9 months from baseline (*p* < 0.05).

EVOO: extra virgin olive oil; LF: low fat; T2DM: type 2 diabetes mellitus; TG: triglycerides; HP diet: high-protein-low-carbohydrate low-calorie diet; S diet: standard protein low-calorie diet; RCT: randomized clinical trial; UCP3: uncoupling protein 3; NPY: neuropeptide Y; MUFA: monounsaturated fatty acids; SFA: saturated fatty acids; APOE: apolipoprotein E; PUFA: polyunsaturated fatty acids; MTNR1B: melatonin receptor 1B; g/day: grams per day; mg: milligrams; mmol: millimoles; L: liter; dL: deciliter. ^a^ Values are reported mean ± SD. ^b^ Values are difference between pre- and post-dietary intervention relative to E3/E3 and were adjusted for baseline, BMI, sex, and age. ^c^ Values are reported adjusted mean (95% CI) change ↓ decrease; ↑ increase; ↔ No significant difference.

## Data Availability

Not applicable.

## Abbreviations

ADA	American Diabetes Association
ALA	α-linolenic acid
CG	Control group
CoQ10	Coenzyme Q10
DHA	Docosahexaenoic acid
EPA	Eicosapentaenoic acid
FDA	Food and Drug Administration
FH	Familial hypercholesterolaemia
G/day	Grams per day
HTG	Hypertriglyceridemia
IG	Intervention group
kcal/day	Kcal per day
LF	Low fat
LPL	Lipoprotein lipase
MCFA	Medium chain fatty acids
mg/day	Milligrams per day
mmol/L	Millimoles per litter
MS	Metabolic syndrome
MUFA	Monounsaturated fatty acids
PPAR	Peroxisome proliferator-activated receptors
PPAR-α	Peroxisome proliferator-activated receptors alpha
PUFA	Polyunsaturated fatty acids
RCT	Randomized clinical trial
SFA	Saturated fatty acids
T2DM	Type 2 diabetes mellitus
TG	Triglycerides
UFA	Unsaturated fatty acids
LDL	Low-density lipoprotein
HDL	High-density lipoprotein
VLDL	Very low-density lipoprotein
HOMA-IR	Homeostatic Model Assessment of Insulin Resistance
